# Nanoscale synthetic biology with innovative medicinal applications

**DOI:** 10.1016/j.fmre.2024.11.006

**Published:** 2024-11-16

**Authors:** Jingsen Ji, Longsong Li, Weisheng Guo, Jing Zhang, Yuying Yao, Haoting Chen, Fangling Liao, Zhaokui Jin, Lu Liu, Jiang Ouyang, Xing-Jie Liang

**Affiliations:** a School of Biomedical Engineering, Guangzhou Medical University, Guangzhou 511436, China; b Department of Gastroenterology, The First Medical Center of Chinese PLA General Hospital, Beijing 100853, China; c Nanomedicine Research Center, The Third Affiliated Hospital of Sun Yat-sen University, Guangzhou 510630, China; d Chinese Academy of Sciences (CAS) Center for Excellence in Nanoscience, CAS Key Laboratory for Biomedical Effects of Nanomaterials and Safety, National Center for Nanoscience and Technology of China, Beijing 100190, China; e College of Nanoscience and Technology University of Chinese Academy of Sciences, Beijing 100049, China

**Keywords:** Nanoscale synthetic biology, Nanocarriers, Assembling systems, Gene editing

## Abstract

The rapid advancement of synthetic biology and nanoscience has given rise to a new research field, known as nanoscale synthetic biology (NSB). This field emphasizes the interactions and coordination within entire biological systems, with the goal of achieving more efficient and controllable biological engineering through nanoscale manipulation. Nanocarriers facilitate the delivery of gene-regulating systems (such as CRISPR, mRNA, siRNA, and plasmids), nanozymes, drugs, and specific nanoprobes, enabling precise control over gene expression, modulation of biological metabolism, and monitoring of synthesized products in various organisms (including cells, bacteria, and viruses). This enables advancements in disease treatment, biological imaging, biocatalysis, and biosensing. Moreover, genetically engineered biological products can be employed in the construction of biomimetic nanocarriers with tailored functionalities, thereby fostering a virtuous cycle between nanoscience and synthetic biology. This review delves into the applications of NSB within the biomedical field, providing insight into common nanocarriers, nano-bio assembling systems, and specific NSB applications. We hope this review aids researchers in deepening their understanding of the interactions between organisms and nanomaterials, ultimately leading to the development of more innovative biological applications, the advancement of scientific technology, and contributions to addressing significant societal challenges.

## 1 Introduction

Synthetic biology is a field that combines knowledge from biology, engineering, and computer science, aiming to design and construct new biological systems or modify existing ones for specific functions. The advent of genetic engineering in the 1970s, marked by milestones such as the manipulation of genes in organisms through recombinant DNA technology, laid the foundation for synthetic biology [1]. The field gradually emerged in the early 21st century, propelled by rapid advancements in biology, computer science, and engineering, particularly in high-throughput genome sequencing. One of its core principles is engineering biological systems to design and construct them like engineers, making biological research more engineering-oriented and controllable for achieving specific functions [2]. Using methods like gene synthesis, genome editing, metabolic engineering, and protein engineering, researchers can modify the genetic programming of biological systems, create new organisms or biosystems, or modify existing ones to exhibit specific functionalities. Synthetic biology uses tools to construct biological systems with specific functions and has broad applications in fields such as medicine, energy, environmental protection, and agriculture [[3], [4], [5], [6]]. In the medical field, synthetic biology can be employed to design and construct new drugs, vaccines, and even applications in cell therapy and tissue engineering. In summary, synthetic biology is an interdisciplinary frontier field that integrates knowledge and techniques from biology, engineering, computer science, and other fields, offering new pathways and possibilities for addressing various significant challenges.

Nanoscience is a field spanning physics, chemistry, materials science, biology, engineering, and other disciplines, involving the design, fabrication, and application of materials, devices, and systems at the nanometer scale [7]. Since the 1980s, with the development of nanoscale imaging techniques like scanning tunneling microscopy and atomic force microscopy [8], researchers have been able to observe and manipulate materials at the nanoscale, leading to rapid advancements in nanoscience-related fields. Its core concept is to design and manufacture materials, devices, and systems with specific functions and properties by controlling and assembling atoms and molecules. Currently, nanoscience has extensive applications in many fields. In materials science, nanoscience can be used to design and fabricate nanomaterials with unique properties, such as inorganic nanoparticles, organic nanoparticles, and biomimetic nanoparticles [9]. These modified nanoparticles find utility in a variety of applications including drug delivery and therapy, biomedical imaging, diagnostic testing, tissue engineering, and regenerative medicine, thereby significantly contributing to the progress of biomedicine [10].

In recent years, the ongoing progress in scientific research has facilitated the convergence of synthetic biology and nanoscience, giving rise to an emerging discipline termed nano-synthetic biology (NSB) [11]. NSB integrates concepts from systems biology, emphasizing the interactions and coordination within entire biological systems. A key challenge in traditional synthetic biology is achieving the efficient synthesis of biomolecules in living organisms. Nanobiotechnology leverages nanomaterials and techniques to enable more precise control of the structure and function of biological systems at molecular levels achieve in vivo biosynthesis. Moreover, nanomaterials possess diverse chemical and physical properties, such as electrical, optical, and magnetic characteristics, which confer a significant advantage in designing multifunctional biological systems [12]. Traditional synthetic biology often relies on the engineering of biomolecules, while nanobiotechnology integrates these modifications with the functional properties of nanomaterials, resulting in more integrated platforms for biosynthesis [13]. In complex intracellular environments, the tools of traditional synthetic biology can be limited by biological barriers or environmental interferences. Nanomaterials, with their excellent biocompatibility and tunable physicochemical properties, are better suited to traverse cellular membranes and modulate intracellular signaling, thereby enhancing the efficiency of biological reactions.

Specifically, scientists can synthesize and modify biological molecules such as DNA, RNA, and proteins using nanoscience to construct organisms with specific functions [14]. Additionally, NSB enables the design, synthesis, and creation of new functionalities and properties that do not exist naturally, resulting in engineered nanocarriers for innovative solutions in fields such as medicine, bioengineering, and so on [15]. Currently, NSB demonstrates innovative potential across multiple application areas. For example, within the medical field, it can be applied to devise enhanced drug delivery platforms, thereby advancing precision medicine [16]. In materials science, it can create nanomaterials with unique properties [[17], [18], [19]]. These innovative applications are poised to drive technological advancements and foster socio-economic development.

**Scheme 1:**
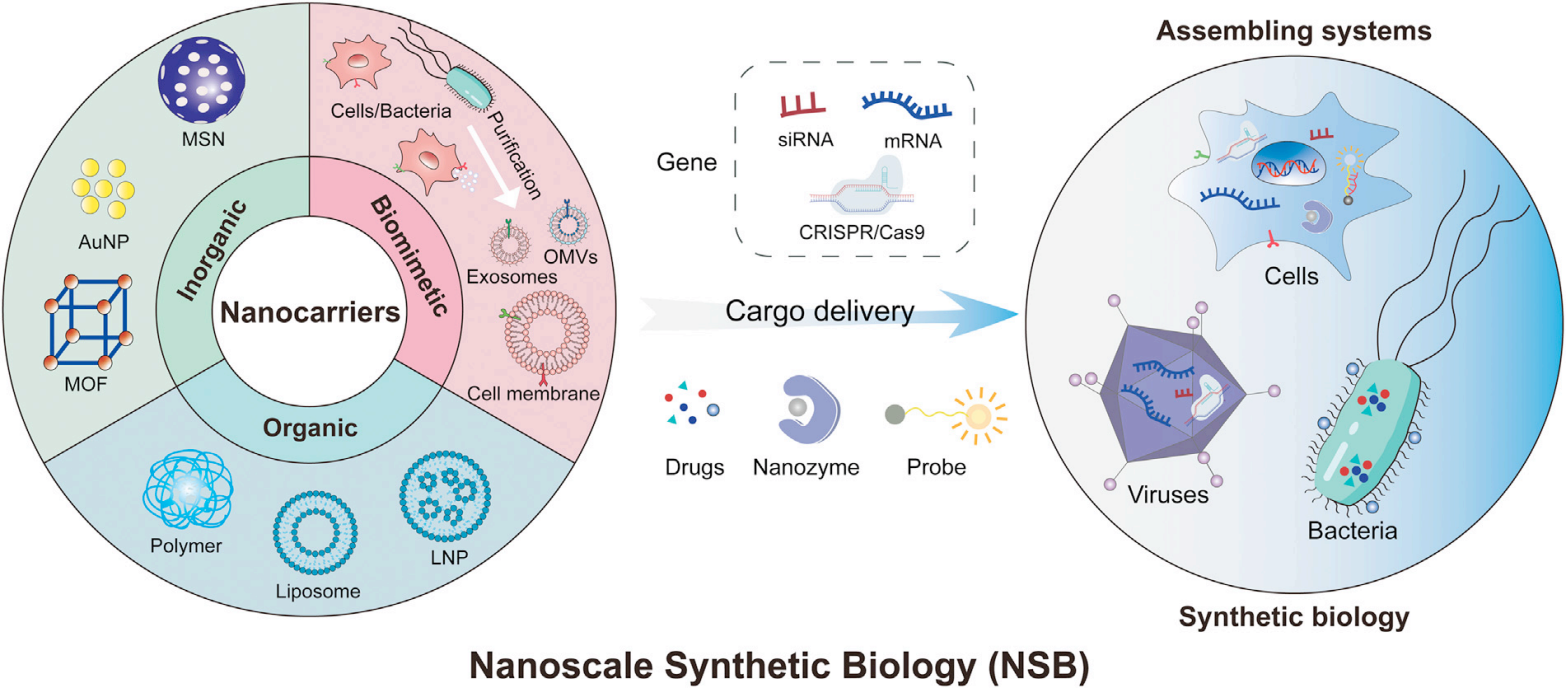
**Illustration of nanocarriers for gene regulation and assembling systems of nanomaterials and biological modules such as cells, bacteria, and viruses that enable precise biosynthetic regulation or obtain nanocarriers with specific functionalities.** MSN, Mesoporous silica nanoparticle; AuNP, Gold nanoparticle; MOF, Metal-organic framework; OMV, Bacterial outer membrane vesicle; LNP, Lipid nanoparticle.

In this review, we first introduced novel nanocarriers that can be applied to regulate biosynthetic processes, including inorganic nanocarriers, organic nanocarriers, and biomimetic nanocarriers [[20], [21], [22]]. These nanocarriers exhibit distinctive characteristics that contribute to drug delivery, gene editing, biological metabolism regulation, imaging, and biosensing [9,23,24]. This interaction enables NSB to draw upon the self-organizing capabilities of biological systems in nature, creating more complex, ordered, stable, and controllable composite systems. Therefore, we also discussed the formation of biological assembling systems involving nanoparticles with cells, bacteria, viruses, etc., enabling precise biosynthetic regulation or obtaining nanocarriers with specific functionalities. NSB achieves precise control over biological systems through bioengineering at the nanoscale, illustrated in Scheme 1. Lastly, we elaborated on the specific applications of NSB in fields such as tumor therapy, regulation of autoimmune diseases, biocatalysis, imaging, and biosensing. In summary, the uniqueness of NSB lies in its focus on nanoscale manipulation and synthesis while integrating principles of biology. This provides scientists with a novel approach to design and construct organisms with specific functionalities, thereby opening up many new research and application avenues.

## 2 Nanocarriers for gene regulation

The advancement of synthetic biology is closely intertwined with the development of novel nanomaterials. In the context of NSB, delivery vehicles refer to using nanocarriers as delivery vectors for genetic or metabolic regulation within organisms, thereby inducing specific phenotypic expressions in target cells [25]. Importantly, nanocarriers can ensure the stability, targeting specificity, and effectiveness of gene delivery. Commonly employed delivery systems include the CRISPR/Cas9 (Clustered Regularly Interspaced Short Palindromic Repeats associated protein 9) gene editing system, mRNA, siRNA, plasmids, among others. Among these, CRISPR/Cas9 stands out as a highly efficient gene editing technology. It utilizes a short segment of specific guide RNA (sgRNA) to guide the Cas9 protein to specific locations within the genome for gene editing [26]. Generally, the CRISPR-Cas9 system can be delivered to target cells in the form of DNA, mRNA, or protein. The Cas9-sgRNA ribonucleoprotein (RNP) complex delivery system offers rapid onset, short duration, and minimal off-target effects, albeit at a higher cost [9].

In addition, nanodevices can serve as signal transduction mediators to regulate gene expression or directly participate in regulating biosynthesis and metabolic catalysis processes as artificial cellular organelles (such as nanozymes) [14,23]. The design of gene circuits enables controlled gene expression, facilitating the advancement of more precise and intelligent drug therapies and gene therapies. During gene transcription, nanomaterials can serve as triggers for various transcriptional and post-transcriptional genes, thereby intervening and regulating gene expression at different levels [27]. For example, through the utilization of external stimuli such as heat, magnetism, light, sound, and more, nanocarriers can function as conduits for signal transduction. They convert these external stimuli into input signals recognizable by gene switches, thereby enabling precise spatiotemporal control over gene expression.

**Fig. 1:**
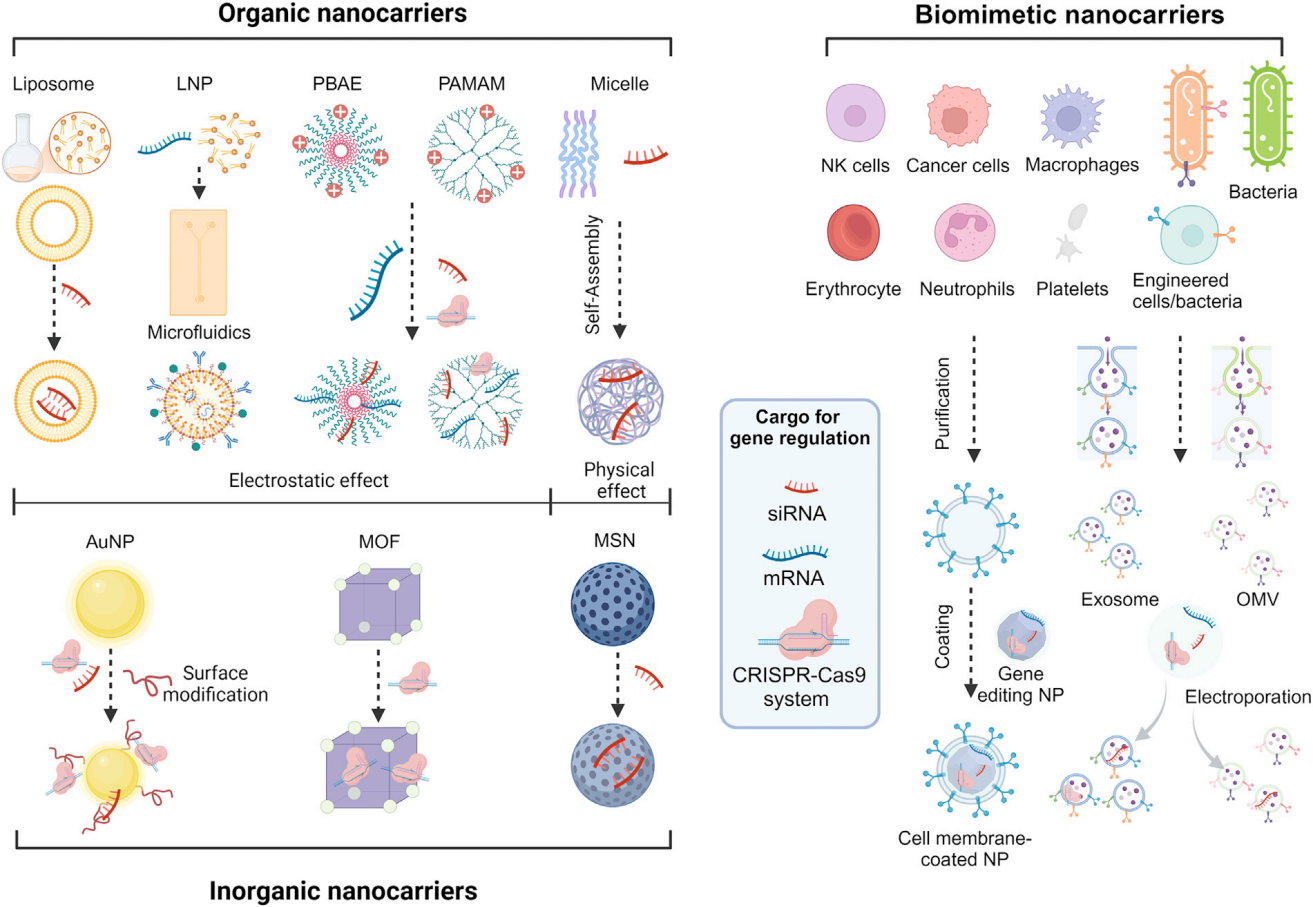
**Illustration of nanocarriers for gene regulation, showing its basic working mechanism and design principle.** PBAE, Poly (beta-aminoester); PAMAM, Polyamidoamine; MSN, Mesoporous silica nanoparticle; AuNP, Gold nanoparticle; MOF, Metal-organic framework; OMV, Bacterial outer membrane vesicle; LNP, Lipid nanoparticle; NP, nanoparticle. Created with BioRender.com/c39u174.

Furthermore, the concept of biomimicry has gained increasing prominence. Scientists are increasingly interested in the unique and stable functions of cell membrane-like materials, which possess complex protein interfaces. By employing a ‘top-down’ strategy, based on natural or genetically engineered biomolecules, highly controllable and functionally specialized nanocarriers can be constructed to further meet diverse needs [28]. Through bioengineering, various organisms (including cells, bacteria, and viruses) can be modified to utilize their biomolecules such as cell membranes, bacterial outer vesicles, and viral capsids. By combining them with multifunctional nanocarriers, specialized biomimetic nano-delivery systems with unique functionalities can be created, applicable to various scenarios [[17], [18], [19]]. This continual progress fosters innovation across various technological domains, presenting novel opportunities in medicine, bioengineering, materials science, and beyond. Fig. 1 summarizes nanocarriers for gene regulation and shows its basic working mechanism and design principle.

### 2.1 Organic nanocarriers

#### 2.1.1 Lipid-based nanocarriers

Lipid-based nanocarriers are nano-sized particle systems used for delivering drugs or other bioactive substances [29]. These carriers are typically composed of lipidic materials such as phospholipids and cholesterol, which can self-assemble to obtain nanoparticles [30]. Extensively researched and utilized within medical and biological realms, these nanocarriers are primarily utilized to augment drug delivery efficacy, enhance drug stability, and reduce drug-related toxicity. Their surface characteristics, size, and drug loading techniques can be precisely tailored to achieve controlled drug release [31,32]. This technology contributes to increasing drug bioavailability within the body while minimizing negative impacts on healthy tissues. Lipid-based nanocarriers hold promising potential for applications in cancer therapy, gene therapy, vaccine delivery, and other fields.

Recently, cationic liposomes have emerged as common non-viral gene delivery vectors, owing to their ability to accommodate the high-density negative charge of nucleic acids. Lipid molecules possess the capability to self-assemble into nanostructures, forming liposomes or lipid nanoparticles. These structures can encapsulate nucleic acid drugs, shielding them from degradation and enhancing their stability in vivo [33]. Lipid-based nanocarriers engage with cell membranes, facilitating the intracellular uptake of cargo molecules through fusion or endocytosis. This characteristic contributes to enhancing the endogenous transport of gene drugs, thereby improving delivery efficiency [29]. Surface modifications can alter the biocompatibility, drug delivery efficiency, and targeting specificity of lipid-based nanocarriers. By conjugating targeting ligands, carriers can selectively interact with specific cell types, improving the precision of gene delivery. For instance, Ke et al. constructed a liver-targeting gene hybrid TKI-fusion lipid (LIGHTFUL), which, through the CRISPR/Cas9 system, can reverse EGFR-mediated drug resistance, thereby effectively enhancing TKI-based therapy for hepatocellular carcinoma (HCC) [34]. LIGHTFUL is coated with lactose-modified membrane fusion lipid layers, facilitating the direct and efficient intracellular transport of internal CDK5 and PLK1-targeted CRISPR/Cas9 plasmids (pXG333-CPs) into HCC cytoplasm, and subsequently into the cell nucleus for high expression. The delivery of pXG333-CP through membrane fusion results in the efficient suppression of CDK5 and PLK1, complete inhibition of EGFR, and enhancement of the anti-HCC efficacy of co-administered TKI lenvatinib. Liposomal carriers can achieve controlled release of gene drugs by adjusting their structure or adding responsive elements, maintaining appropriate drug concentrations and mitigating side effects [35].

Lipid nanoparticle (LNP) is a novel nanocarrier system developed based on liposomes, which, compared to traditional liposomes, possess smaller size, broader application scope, and more diverse structural forms. Typically, LNPs consist of four components with distinct functions, including permanently charged cationic or ionizable cationic lipids, phospholipids, cholesterol, and polyethylene glycol (PEG) lipids LNPs comprise four components with distinct roles, including cationic or ionizable cationic lipids, cholesterol, phospholipids, and polyethylene glycol (PEG) lipids, each contributing to their functionality [36]. As gene delivery carriers in gene therapy, lipid nanoparticle carriers play a crucial role in efficiently delivering nucleic acids such as DNA, mRNA, or siRNA to target cells, thereby regulating gene expression [37]. Two commercially available mRNA vaccines for SARS-CoV-2 are indeed ionizable lipid nanoparticles. The advancement of LNPs has undergone rapid progress, witnessing the emergence of numerous novel LNPs endowed with specific functionalities for nucleic acid delivery, alongside the traditional LNPs (Table 1).

**Table 1:** **Representative studies of LNPs in NSB**.

LNP	Cargo	Function	Administration	Reference
Traditional LNP	uniSTING-mRNA	promotion of DC maturation and antigen-specific CD8 T-cell responses	i.m.(tumor)/ i.v.	[41]
	siRNA targeting the PU.1 transcription factor	reduction of neuroinflammation	i.c.	[42]
	nonintegrative and safe nucleoside-modified VEGFA mRNA	promotion of liver repair	i.o.	[43]
Supramolecular LNP	mRNA encoding tumor antigen and a TLR7/8 agonist	promotion of DC maturation and antigen presentation	i.m.	[44]
Activating LNP	CD3 and CD28 antibody fragments and CAR mRNA	promotion of T cell activation, transfection and differentiation	i.v.	[45]
Synergistic LNP containing two different ionizable lipids	VZV glycoprotein E -encoding mRNA	induction of robust humoral and cellular immune responses	i.m.	[46]
Multiply adjuvanted LNP	mRNA encoding for the fusion protein (SARS-CoV-2)	enhanced activation of innate and adaptive responses	i.m./i.n.	[47]
Adjuvant lipidoid-substituted LNP	SARS-CoV-2 mRNA	enhanced activation of innate and adaptive responses	s.c.	[48]
Noncationic thiourea LNP	OVA mRNA	spleen-targeting delivery and induction of robust humoral cellular immune responses	s.c./i.v./i.m.	[49]

i.v., intravenous; i.m., intramuscular; i.m.(tumor), intratumoral; i.c., intracisternal; i.n., intranasal; s.c., subcutaneous; i.o., retroorbital.

By altering the functional groups of LNPs, their physicochemical properties and transfection efficiency can be influenced. Milan et al. demonstrated that surface modification of LNPs by conjugating PEG-lipids with amine (LNPa), carboxylic acid (LNPz), and carboxyl ester (LNPx) functional groups, while loading mRNA, resulted in enhanced gene expression in photoreceptors by LNPx and LNPz, whereas LNPa and unmodified neutral LNPs only retained transfection in retinal pigment epithelium [38]. Natural products, owing to their excellent biocompatibility, are also commonly employed as carriers for drug delivery. Hou et al. developed a range of vitamin-derived LNPs designed for delivering antimicrobial peptide and cathepsin B mRNA [39]. Five vitamin-derived lipids, namely V_B3_-lipid, V_C_-lipid, V_D_-lipid, V_E_-lipid, and V_H_-lipid, were synthesized through carboxylic acid esterification with amino-containing tertiary amines. The lipids obtained can undergo ionization under acidic conditions, thus offering positive charges to interact with mRNA. LNPs based on V_C_-lipid exhibited 20 times higher efficacy compared to other LNPs. Furthermore, exploring novel cationic or ionizable lipids holds significant promise alongside optimizing LNP formulations to augment transfection efficiency. Cationic or ionizable lipids generally comprise a hydrophobic tail, an amino head, and a linker connecting them. Therefore, introducing various functional groups of the lipid can enhance transfection efficacy. For instance, Zhang et al. synthesized an ionizable lipid library with dendritic amino nucleus as the lipid head and functionalized N1, N3, N5-tri(2-aminoethyl) benzene-1,3,5-tricarboxamide as the lipid tail [40]. The study revealed that lipid unsaturation could potentially result in decreased transfection efficiency. Conversely, LNPs with lipid branched ester chains demonstrated excellent transfection efficacy, likely attributable to variances in their liver clearance rates and degradation rates.

#### 2.1.2 Polymer nanocarriers

Polymer nanocarriers are important in nucleic acid delivery, which is a crucial research direction in synthetic biology. These carriers efficiently deliver nucleic acid drugs to target cells, thereby regulating gene expression for disease treatment or gene function research. Although not as advanced clinically as LNPs, polymer nanocarriers exhibit promising potential as delivery systems, typically comprising biodegradable amine-containing polymers that can self-assemble with RNA.

Polyethyleneimine (PEI) has been considered the ‘gold standard’ for gene delivery in the past. Amine-containing PEI not only enhances the membrane affinity of polymer nanoparticles and improves cellular uptake but also induces the endosomal effect [50]. In the acidic environment of endosomes, polymers containing amine groups undergo protonation, leading to the continuous influx of protons into the endosome/lysosome. To maintain electrolyte balance, chloride ions are actively pumped into the endosome/lysosome, resulting in an accumulation of ions and an increase in osmotic pressure within these organelles. This elevated osmotic pressure ultimately leads to the rupture of the endosome/lysosome, facilitating the release of the polymer carrier—a phenomenon commonly referred to as the ‘proton sponge effect’ [51]. However, cationic polymers with high positive charges can bind to serum proteins and red blood cells, leading to membrane rupture [52]. Therefore, their application is limited due to safety concerns.

Poly (beta-aminoester) (PBAE) is another type of cationic polymer composed of ester bonds. Due to its ease of synthesis and lower cytotoxicity compared to PEI, PBAE demonstrates elevated transfection efficacy, showing significant promise in the domain of gene delivery [53,54]. Currently, materials based on poly(ε-caprolactone) PBAE polymers, PBAE with oligopeptide terminal modifications, and hyperbranched PBAE have been developed to augment the payload capacity and delivery efficiency of nucleic acids [[55], [56], [57]].

**Fig. 2:**
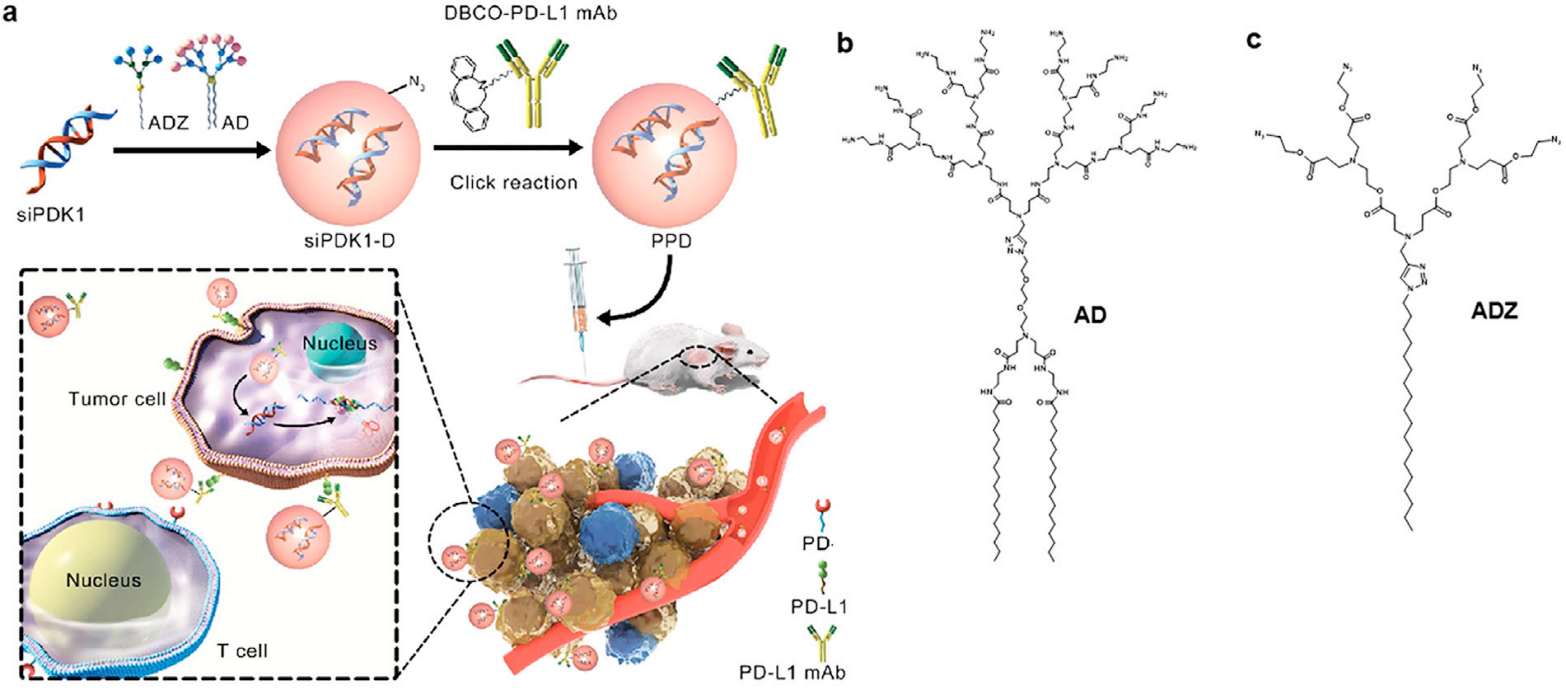
**Schematic representation illustrating the formation of the siPDK1-PD-L1 mAb-dendrimer complex (PPD).** (a) The engineered PPD effectively shields siPDK1 from degradation and facilitates its delivery into tumor cells, resulting in the downregulation of PDK1, which significantly impedes cancer cell proliferation, adhesion, migration, and invasion, all with minimal toxicity. (b,c) Chemical structures are presented for the amphiphilic dendrimer AD with amine terminals for siRNA transport (b) and the amphiphilic dendrimer ADZ with azido terminals designed for click conjugation with DBCO-modified PD-L1 antibody (c) [59]. Reprinted with permission.

Dendrimers are a type of synthetic polymer known for their spherical and highly branched structures, which feature a high density of surface functional groups [58]. Within this class, polyamidoamine (PAMAM) dendrimers, in particular, possess residual amine groups that can undergo electrostatic interactions with anionic groups present on proteins or RNA. This characteristic facilitates the effective delivery of ribonucleoproteins (RNPs) or RNA molecules into cells, thereby enabling intervention and gene editing. Moreover, by introducing specific targeting molecules such as antibodies, peptides, or other biomolecules onto the surface of dendrimers, the selective interaction of these carriers with particular cell types can be enhanced. This strategy serves to enhance the targeted delivery efficiency of nucleic acids. Zhang et al. illustrated the conjugation of the dendrimer ADZ with the dibenzocyclooctyne-modified PD-L1 antibody using click chemistry (Fig. 2) [59]. Through electrostatic adsorption, siPDK1 was loaded onto PD1-modified PAMAM (PPD), leading to the formation of a self-assembled dendrimer nano-system. PPD effectively suppressed both PDK-induced glycolysis and immune reactions linked with the PD-1/PD-L1 pathway, thus efficiently impeding breast cancer growth and metastasis. mRNA can also be coated within the core of nano micelles [60]. An example includes the self-assembly of vitamin E succinate-modified polyethyleneimine copolymers into polymer micelles, which efficiently encapsulate and protect mRNA molecules, exhibiting effective vaccine delivery in vivo [61]. Moreover, siRNA can be linked with polymers via click chemistry. Jiang et al. constructed a novel non-cationic siRNA micelle composed of siRNA-disulfide-poly(N-isopropyl-acrylamide) (siRNA-SS-PNIPAM) diblock copolymers via self-assembly [62]. These siRNA micelles not only exhibit efficient drug loading capacity, prolonged circulation time in blood, excellent cellular uptake, and effective siRNA release but also demonstrate robust BBB permeability. In a U87 orthotopic glioma mouse model, this novel RNA interference (RNAi) nanomedicine significantly inhibited tumor growth and extended the survival of mice.

The study on polymer-based nanocarriers for nucleic acid delivery aims to surpass the constraints associated with free nucleic acid delivery, improve nucleic acid stability, enhance cellular uptake efficiency, and target specificity, thus providing more potent tools for gene editing. However, it is essential to acknowledge that the intricacy and safety of carrier design continue to be pivotal focal points in this area of research.

### 2.2 Inorganic nanocarriers

Gold nanoparticles (AuNPs) are nano-sized particles composed of gold atoms, typically ranging in size from 1 to 100 nanometers. AuNPs exhibit excellent stability and biocompatibility, and they are easily amenable to surface modifications, making them ideal carriers for gene therapy [63]. The surface of AuNPs can be chemically modified by introducing different functional groups, allowing precise control over the carrier properties. Moreover, targeting molecules can be incorporated to achieve specific delivery to particular tissues or cells [64]. AuNPs can efficiently adsorb nucleic acid molecules such as DNA, RNA, or siRNA, providing a stable means to protect nucleic acids from degradation and enhance their stability in vivo. Furthermore, to deliver RNP complexes to macrophages via systemic administration, Lee et al. prepared an arginine-modified gold nanoparticle delivery system [65]. Following intravenous injection, the AuNP delivery system primarily accumulated in the livers and spleens of mice, with a gene insertion deletion frequency of up to 8% detected in tissue macrophages. AuNPs possess the capability to absorb specific wavelengths of light, leading to photothermal effects [66]. This property can be exploited for localized heating, achieving controlled drug release and photothermal therapy. For instance, Wang et al. integrated stable, high-drug-loading lipid nanoparticles with AuNPs capable of controlled release in vitro to construct a lipid-coated AuNPs-plasmid for targeting the melanoma site [67]. The AuNPs, with a diameter of 20 nm, were initially synthesized and subsequently electrostatically bound to negatively charged plasmids to create a polymer core, which was further encapsulated with cationic liposomes and modified with PEG2000-DSPE to form the lipid-coated AuNPs-plasmid. The lipid shell stabilized the overall structure and facilitated cellular internalization. The AuNP core served as both a carrier and a responder to photothermal conditions, enabling controlled release of the plasmid. Additionally, AuNPs can also be used for imaging and delivery process monitoring due to their distinctive surface-enhanced Raman scattering and localized surface plasmon resonance effects [68]. AuNPs hold promise as gene delivery carriers, providing new avenues to enhance delivery efficiency and precision. Nevertheless, additional research is warranted to explore their toxicity, biodistribution, and other factors to ensure their safety and viability in biomedical applications.

Metal-organic frameworks (MOFs) are organic-inorganic hybrid crystalline porous materials composed of inorganic metal ions and organic molecules, while Zeolitic Imidazolate Frameworks (ZIFs) are a subclass of MOFs consisting of tetrahedral coordinated transition metal ions and imidazolate linkers [69]. Metal ions in MOFs can interact with proteins or RNPs through a combination of coordination and ionic interactions. To date, ZIF-8 and ZIF-90 have been employed for intracellular RNP delivery [70]. RNPs can be encapsulated within ZIF during the formation process, and the imidazole moiety in ZIF can facilitate RNP complex endosomal escape through a pH-buffering mechanism. ZIF-8/RNP nanoparticles achieved 30% targeted EGFP knockout in Chinese hamster ovary cells, while ZIF-90/RNP complexes knocked out 40% of the GFP gene in HeLa cells. Hybrid nanoparticles comprised of ZIFs and silica exhibited notable efficacy in vivo delivery of RNPs [71]. MOFs can not only be utilized for gene delivery but can also combine with other functional components such as drugs, imaging agents, or photosensitizers to form multifunctional delivery platforms [72]. Wu et al. synthesized DOX-encapsulated ZIF-8/SrSe nanozymes, which exhibit notable glutathione (GSH) oxidase-like activity [73]. This nano-system synergistically induces apoptosis in tumor cells by depleting GSH and concurrently releasing the chemotherapeutic drug DOX. Despite exhibiting many advantageous properties in drug delivery, MOFs still face challenges such as biocompatibility, toxicity, and stability. Therefore, researchers are continuously striving to improve the design and synthesis methods of MOFs to enhance their efficacy in gene delivery and further advance their biomedical applications.

Mesoporous silica nanoparticles (MSNs) present opportunities for high-capacity cargo loading owing to their exceptional stability, versatility in modification, and extensive internal as well as external surface areas. As carriers, MSNs can encapsulate nucleic acid molecules within their pores, forming complexes. These complexes can protect nucleic acid molecules and accomplish targeted delivery in vivo through surface modifications, making them a promising gene delivery carrier [74]. Liu et al. designed a delivery system based on MSNs, by loading a sgRNA targeting programmed death-ligand 1 and axitinib, for the treatment of melanoma in mice [75]. This combination therapy led to a 50% reduction in tumor volume and a 20% increase in survival rate of B16-F10 melanoma-bearing mice. It is crucial to acknowledge that while MSNs exhibit some advantageous properties in nucleic acid delivery, further research is needed to fully understand their biocompatibility and toxicity.

### 2.3 Biomimetic nanocarriers

Artificially designed organic or inorganic carriers, despite being functional with various modifications for different purposes, are inevitably recognized and cleared by the body's immune system as foreign entities. Biomimetic nanocarriers refer to nano-scale materials derived from biological sources with specific biological functions obtained through extraction and purification processes [76]. Cell membranes sourced from biological organisms typically exhibit excellent biocompatibility, thereby enhancing their circulation half-life [77,78]. These biomimetic nanocarriers inherit some functional characteristics of living organisms, allowing them to better meet practical needs [79]. it becomes feasible to design, synthesize, and tailor molecules, cells, or tissues sourced from living organisms, yielding biologically derived nanomaterials endowed with specific functionalities, by using techniques from synthetic biology, such as genetic engineering and protein engineering. These materials find diverse applications across fields including medicine, materials science, and bioengineering. Moreover, during the onset of diseases, local microenvironments frequently undergo alterations. Biomimetic nanomaterials derived from biological sources can capitalize on receptor-ligand interactions or chemotaxis triggered by these changes. Consequently, they enhance targeting or homing effects, augmenting the accumulation of gene drugs at disease sites while mitigating off-target effects.

#### 2.3.1 Exosomes

The majority of eukaryotic cells release extracellular vesicles (EVs) as pivotal mediators of intercellular communication, enabling information exchange through diverse mechanisms, including the secretion of chemokines and growth factors [80]. Exosomes, with diameters typically ranging from 30 to 100 nm, represent a class of naturally secreted cell-derived EVs. They play crucial roles in intercellular communication by virtue of their cargo, which includes cell adhesion molecules, ligands on their membranes, or encapsulated contents [81]. In comparison with synthetic carriers, EVs offer numerous advantages as natural carriers. For example, their high biocompatibility affords them prolonged half-lives in vivo [82]. Moreover, Exosomes retain lipids and proteins reflective of their parental cells, they exhibit preferential interactions and fusion with cell types of origin, facilitating more precise gene delivery to target cells or tissues. Additionally, Exosomes demonstrate lower immunogenicity compared to some viral vectors, thus reducing the occurrence of immune responses.

Cas9 RNPs loaded into purified exosomes isolated from hepatic stellate cells can be transported to liver tissue [83]. Wan et al. prepared sgRNAs targeting p53 and apoptosis regulators, cyclin E1, and lysine acetyltransferase 5, which were then coupled with Cas9 to construct RNPs. These RNPs were efficiently transported to the liver via exosome carriers, enabling effective gene editing [84]. Studies indicate that the exosome-RNP genome editing system is a perfect vector for targeted treatment of liver illnesses including cirrhosis and fibrosis because of its capacity to target the liver. Bu et al. successfully controlled inflammation in atherosclerotic plaques in mice by delivering engineered IL-10 mRNA via exosomes [85]. Moreover, there is a greater delivery efficiency and less cytotoxicity when exogenous miRNA-155 mimics or inhibitors are delivered to hepatocytes or macrophages via exosomes produced from B cells [86].

However, the functionality of conventionally acquired exosomes is also limited, and it is worth considering engineering modifications to obtain specific functions. For example, Liu et al. designed exosomes by expressing the ANG-TRP-PK293 peptide in HEK1T cells [87]. The ANG-TRP-PK293 peptide confers BBB penetrance and glioblastoma targeting ability to the exosome carrier, enhancing its capacity to kill glioma cells. Similarly, engineered exosomes doubly modified with Angiopep-2 (Ang) and trans-activating transcriptional activator (TAT) peptides exhibit dual BBB and glioblastoma targeting, enabling efficient delivery of Cas9 protein/sgRNA complexes into glioblastoma cells for high-efficiency gene editing [88]. This nanosystem disrupts glutathione synthesis through depletion of glutathione synthetase, leading to inactivation of GPX4 and accumulation of iron, thereby enhancing ferroptosis post-radiotherapy. These findings underscore the significant potential applications of exosomes in the biomedical field, including gene and medication delivery carriers.

#### 2.3.2 Cell membrane-coated nanoparticles

Cell membrane-coated nanoparticles are nanostructures inspired by the structure of biological cell membranes, characterized by mimicking both the function and structure of natural cell membranes [13]. This design combines the flexibility of artificially synthesized nanoparticles with the natural properties of biological membranes to enhance the biocompatibility, stability, and targeting capability of the carriers, while reducing immunogenicity and toxicity in vivo [89]. By introducing targeting molecules (such as antibodies, ligands, etc.) onto the membrane coating, cell membrane-coated nanoparticles can accurately deliver drugs or other bioactive substances to specific cells or tissues [79]. Furthermore, owing to the flexibility and adaptability of natural membranes, this nano-system can more easily penetrate biological barriers [17]. The cell membrane-coated nanosystems can be functionalized as required, exhibiting various functionalities including drug delivery, immune modulation and targeted therapy, holding promise for widespread applications in biology and medicine.

**Fig. 3:**
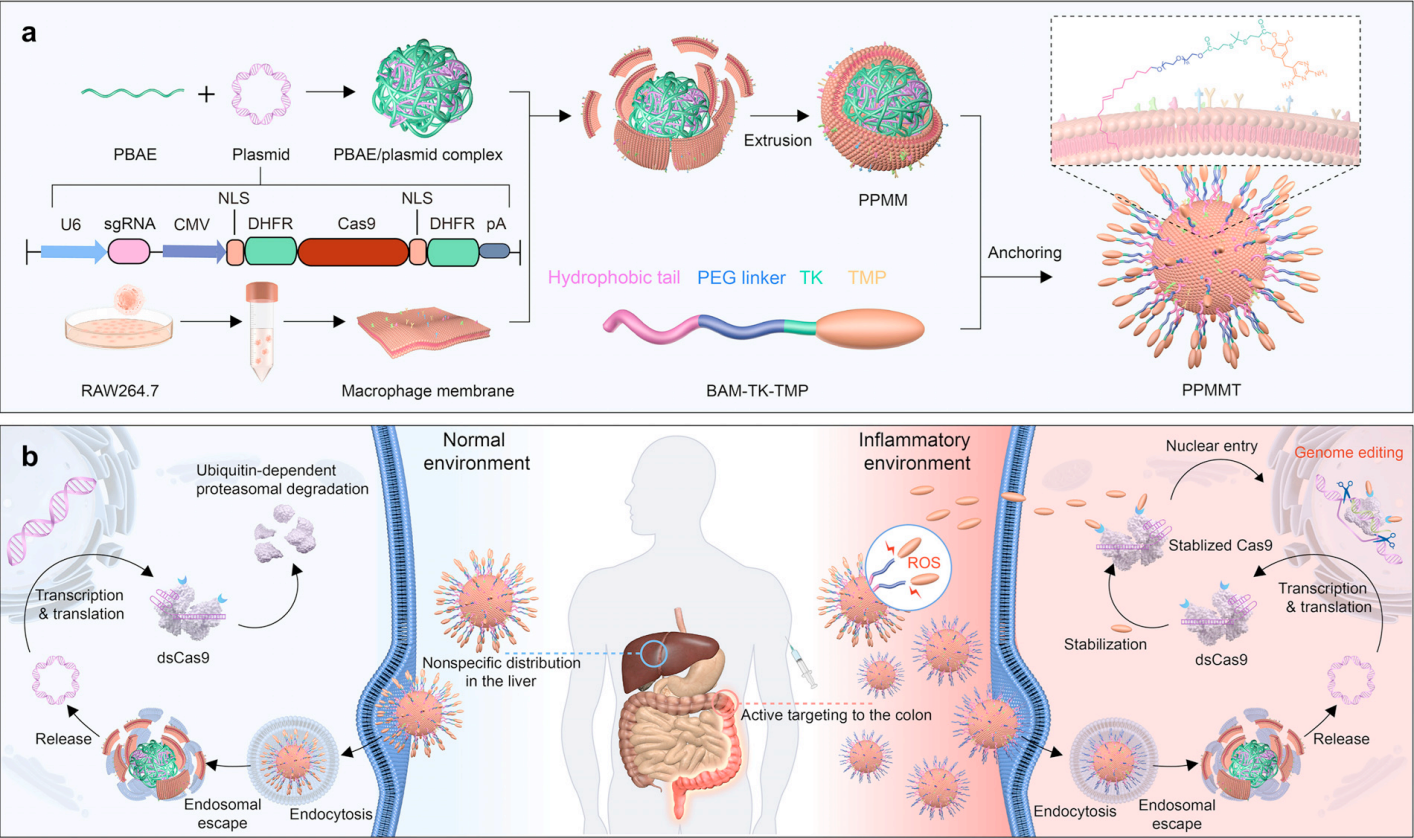
**Schematic overview of targeted delivery and inflammation-specific gene editing using the NanoProCas9 system.** (a) Preparation of the PPMMT complex involves multiple stages. Initially, a plasmid encoding the dsCas9 sequence linked to DHFR domains is designed and subsequently complexed with cationic PBAE polymer to form PBAE/plasmid complexes. Following this, the polyplexes undergo coating with membranes derived from RAW264.7 cells via extrusion to yield membrane-coated polyplexes (PPMM). Finally, a stabilizing molecule (TMP) for dsCas9 is conjugated to OE-PEG using a ROS-sensitive thioketal linker, resulting in BAM-TK-TMP, which is attached to the PPMM membrane to create PPMMT. CMV stands for cytomegalovirus. (b) Representation of inflammation-specific targeting and genome editing within inflamed colon lesions. PPMMT selectively targets inflamed colon areas through MMs, facilitating TMP release via thioketal linker cleavage in the inflammatory microenvironment. This allows the plasmid delivered by PPMMT to be translated into dsCas9, stabilized by TMP, thereby restoring genome-editing functionality. In non-inflamed tissues, dsCas9 undergoes proteasomal degradation via ubiquitination, reducing off-target genome editing despite broad distribution of PPMMT nanoparticles [95]. DHFR, dihydrofolate reductase; PBAE, poly(β-amino ester); TK, thioketal linker; TMP, trimethoprim. Reprinted with permission.

Currently, various cell membrane types have been used for effective and targeted delivery. For example, red blood cell membranes are utilized to prolong the circulation half-life of nanoparticles [90], platelet membranes are employed for targeting damaged blood vessels or tumors [91,92], neutrophil membranes are capable for inflammation targeting [93], and tumor cell membranes serve purposes in tumor targeting or vaccine preparation [94], among others. Yan et al. prepared cationic PBAE complexes with plasmids encoding the CRISPR system as the core, with the macrophage membrane covering the surface of the PBAE/pDNA complexes (Fig. 3) [95]. Additionally, reactive oxygen species (ROS)-responsive elements were integrated onto the external surface of the membrane. In this biologically inspired nanomaterial, macrophage membrane targeted inflammatory lesions, facilitating nanoparticle internalization and release under high ROS levels. Liu et al. designed a neutrophil membrane-coated ZIF-8 nano-delivery platform (AM@ZIF@NM) for loading anti-miRNA-155 oligonucleotides into endothelial cells within atherosclerotic lesions. Here, the interaction between the CD18 protein on the neutrophil membrane surface and intercellular adhesion molecule-1 on the endothelial cell surface mediated plaque targeting. Effective delivery of anti-miR-155 downregulated miR-155 expression, thereby modulating the expression of related target genes, suppressing inflammation at the lesion site, and alleviating atherosclerosis [96]. Similarly, engineered cell membranes can provide other functions, such as overexpression of tumor necrosis factor-alpha (TNF-α) receptors on macrophage membranes via plasmid transfection. Extracted cell membranes are used to load mRNA for arthritis therapy (MR@P-mPTEN). This biomimetic system competitively binds excess TNF-α in vivo and activates the PTEN pathway, thereby inhibiting synovitis and joint damage [97].

Collectively, both EVs and cell membrane-coated nanoparticles can encapsulate drug payloads within their internal core materials, allowing for modulation of membrane properties and structure to achieve functionalities such as controlled release, targeted delivery, and biodegradation.

#### 2.3.3 Nanocarriers derived from bacteria

Bacterial outer membrane vesicles (OMVs) are small vesicles released from the outer membrane of Gram-negative bacteria. They are typically enclosed by a double-layered lipid membrane and enriched with various pathogen-associated molecular patterns derived from bacteria, such as lipopolysaccharides, peptidoglycans, proteins, DNA fragments, etc. Therefore, they can efficiently stimulate the immune system and are often used as vaccines or vaccine carriers against pathogenic microorganisms [98]. OMVs hold significant research and application value in the areas of medication delivery, immunology, and bacterial physiology.

**Fig. 4:**
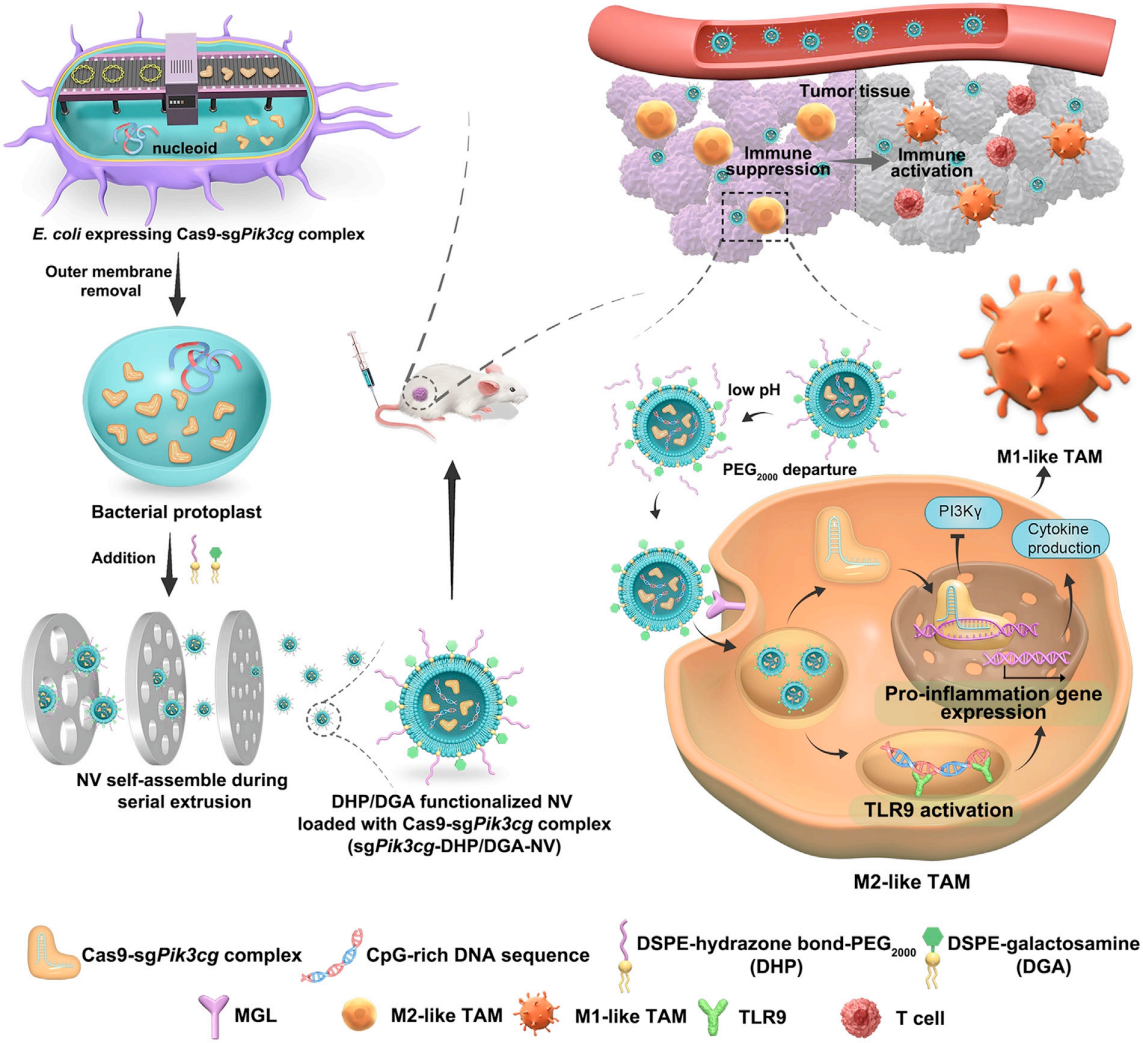
**In this illustration, the authors outline the creation of sg*Pik3cg*-DHP/DGA-NVs for targeted genome editing in TAMs to enhance anti-tumor effects.** The process begins with the construction of *E. coli* expressing the Cas9-sg*Pik3cg* complex. Subsequently, the bacterial outer membrane, which possesses high endotoxicity, is removed. This results in the formation of sg*Pik3cg*-DHP/DGA-NVs, which encapsulate a substantial amount of Cas9-sg*Pik3cg* ribonucleoproteins (RNPs) and CpG-rich genomic DNA. These nanovesicles (NVs) are produced through a series of extrusion steps and are further modified with a pH-responsive phospholipid derivative (DHP) and a phospholipid derivative targeted specifically to TAM (DGA). Upon intravenous injection, sg*Pik3cg*-DHP/DGA-NVs accumulate in tumor tissues due to their prolonged circulation capability and the enhanced permeability and retention (EPR) effect. Within the acidic microenvironment of the tumor, PEG2000 separates from DHP, triggering the recognition and internalization of DGA-functionalized NVs by TAMs via macrophage galactose-type lectin (MGL) receptor-mediated endocytosis. This process enables TAM-specific genome editing of *Pik3cg* and activation of toll-like receptor 9 (TLR9) in vivo, resulting in the reprogramming of M2-like TAMs into an anti-tumor M1-like phenotype and facilitating tumor immunotherapy [101]. Reprinted with permission.

Li et al. harnessed genetically engineered OMVs as a carrier for mRNA delivery, where the surfaces were tailored with RNA binding protein L7Ae and lysosomal escape protein listeriolysin O (OMV-LL) [99]. OMV-LL-mRNA was taken up by dendritic cells, and it enabled listeriolysin O-mediated endosomal escape to transport mRNA antigen. Surprisingly, OMV-LL-mRNA not only completely regressed a colon cancer model by 37.5% and significantly suppressed the growth of melanoma, but it also induced a lasting immunological memory in mice. Besides their immunostimulatory and inflammatory attributes, OMVs exhibited prowess as proficient drug delivery carriers. Shi et al. pioneered the development of non-inflammatory magnetic OMVs from *Escherichia coli* via CRISPR/Cas9-mediated gene knockout targeting inflammatory lipid A acyltransferase synthesis, followed by treatment with iron oxide magnetic nanoparticles [100]. These magnetic OMVs, loaded with the antibiotic ceftriaxone and sonosensitizer meso‑tetra(4-carboxyphenyl) porphyrin (TCPP), were guided magnetically across the BBB. Upon low-frequency ultrasound activation, TCPP generated ROS synergistically with ceftriaxone, conferring antibacterial efficacy and yielding significant therapeutic outcomes in a meningitis model of mice. Zhao et al. leveraged native bacterial vesicles derived from bacterial cytoplasm loaded with Cas9-sgRNA RNP targeting *Pik3cg*, a pivotal molecular switch for macrophage polarization, along with bacterial CpG-rich DNA fragments as potent TLR9 ligands (Fig. 4) [101]. These engineered nanovesicles, modified with pH-responsive PEG-conjugated lipids and lactosamine-conjugated lipids, were engineered for targeting tumor-associated macrophages (TAMs). By stabilizing the M1-like phenotype in TAMs, this novel approach reshaped the tumor microenvironment and hence inhibited tumor growth in mice.

Currently, biomimetic nanocarriers have made considerable advancements, yet their clinical translation faces certain challenges. For example, cell membrane-derived nanovesicles and EVs carry donor cell information, while the immunogenicity and biocompatibility of bacterial OMVs also require thorough evaluation. Furthermore, addressing issues related to achieving scalability in production and ensuring consistency in preparation is imperative.

In the fields of gene editing and biosynthesis, the selection and design of nanocarriers are of paramount importance. Different types of carriers can significantly impact delivery efficiency, the precision of gene editing, and the resulting biological functions. When evaluating these carriers, it is crucial to consider factors such as stability, targeting capability, drug-loading capacity, and biocompatibility, while optimizing the design based on specific application requirements. The delivery of gene editing tools, such as CRISPR-Cas9, requires efficient penetration of the cell membrane and subsequent entry into the nucleus or cytoplasm to achieve its editing function. Nanocarriers like cationic liposomes, LNPs, and cationic polymers are frequently used for delivering DNA or RNA due to their ability to form complexes with negatively charged nucleic acids. The choice of such carriers is typically based on their delivery efficiency and compatibility with different cell types.

A current development direction is to enhance the targeting capability of carriers to ensure that gene editing occurs specifically in target cells, thus avoiding off-target effects. For example, researchers have explored surface modifications using specific ligands or antibodies, which enable the carriers to recognize and bind to particular cell surface receptors, thereby achieving targeted delivery. This strategy is particularly important for treating diseases in specific tissues, such as disorders of the central nervous system or for liver-targeted therapies.

The biocompatibility of nanocarriers during in vivo applications directly affects the safety of therapeutic interventions. While carriers such as LNPs and cationic liposomes exhibit relatively good biocompatibility, they can still provoke immune responses. Hence, reducing immunogenicity remains a major challenge in current design strategies. Recently, exosomes and cell membrane-coated nanoparticles have become research hotspots due to their low immunogenicity and superior biocompatibility, which can significantly extend their circulation time in the body. In gene editing applications, these natural nanocarriers enable more stable delivery of editing tools, reducing the risk of clearance and improving therapeutic efficacy. However, the low yield and complex purification of exosomes remain significant bottlenecks. Future research could focus on leveraging cell factories or developing artificial nanosystems that mimic the characteristics of exosomes, aiming to lower costs and increase production efficiency.

In gene editing and biosynthesis applications, the multifunctionality of delivery carriers can significantly enhance therapeutic outcomes. For instance, nano micelles and MOFs can transport gene-editing tools while simultaneously delivering small-molecule drugs, thus achieving a synergistic effect between gene editing and drug therapy. AuNPs and MSNs are also widely used in the co-delivery of multiple drugs due to their tunable surface chemistry. These carriers can regulate cellular metabolic pathways while performing gene editing, leading to improved synergistic therapeutic effects in cancer treatment. This multifunctional design can further be applied to other scenarios, such as regulating gene expression while simultaneously activating immune responses, which is valuable for cancer immunotherapy. Consequently, future research should focus on developing more precise and intelligent carriers that can control drug release and gene-editing activity in response to changes in the cellular microenvironment.

The application of stimuli-responsive nanocarriers in gene editing is a key area of advancement. These carriers can release gene-editing tools precisely in response to specific stimuli in the tumor microenvironment, such as pH changes, redox conditions, or enzymatic activity. MOFs and certain modified cationic polymers have shown great promise in this field. For example, MOFs can be engineered by adjusting the combination of their metal nodes and organic linkers to respond to acidic or oxidative conditions, enabling site-specific delivery in tumor tissues. This microenvironment-responsive property is crucial for enhancing the precision of gene editing and reducing toxicity to non-target tissues, thereby improving therapeutic outcomes.

The future design of nanocarriers will focus on modularity and programmability, allowing for the customization of carriers by adjusting their individual components according to specific application needs. This approach significantly enhances the adaptability and range of applications for nanocarriers. By utilizing NSB, more sophisticated nanocarriers can be engineered—carriers that not only deliver gene-editing tools but also regulate molecular processes within cells, even potentially repairing intracellular metabolic pathways. Key research areas include the development of combination carriers, improving the safety and targeting capability of existing carriers, and leveraging synthetic biology tools to optimize carrier design. These advancements aim to accelerate the translation of these technologies into clinical applications, ultimately providing more precise and effective therapies for patients.

## 3 Assembly of nanomaterials and biological modules

Traditional synthetic biology is limited by the lack of effective in vivo modification methods, often relying on standardized biological components (such as genes and proteins) for recombination to amplify target protein products in vitro or construct new biological systems to meet various needs. With the advancement of NSB, in vivo biological modifications have become feasible, including the organic combination of gene-editing nanocarriers with target cells, as well as the direct integration of functional nanosystems with living organisms to mimic biological activities, or endow new functions. In this review, we propose nano-bio assembling systems, which refers to the integration of nanoscale materials with biological components to create functional systems.

For instance, nanomaterials are employed as carriers for the precise delivery of drugs and nucleic acids for therapeutic purposes; biomimetic nanoenzymes are designed to modulate biosynthesis and metabolic pathways within organisms; and nanosensors are developed for monitoring physiological parameters or disease biomarkers in vivo. In addition, engineered bacteria modified in vitro can carry multifunctional nanomaterials to perform complex functions, such as achieving targeted delivery and activating the immune system. virus-like nanoparticles can mimic viral infection behaviors to transport relevant genes. Therefore, by leveraging the distinct characteristics of biological entities such as cells, bacteria, and viruses, assembling nanomaterials with biological modules can construct structured and functionally controllable bio-nanohybrid systems, thereby expanding the applications of biotechnology and nanotechnology.

### 3.1 Assembling systems of nanomaterials and cells

Cells, as the fundamental units of the human body, hold significant research value due to their wide range of essential functions, such as metabolism, energy conversion, cell division, signal transduction, protein synthesis, motility, apoptosis, and intercellular communication [102]. These functions interact synergistically to maintain normal physiological states, forming the complete biological structure and functional systems of living organisms. The advancement of nanotechnology has enabled precise regulation of biosynthetic processes within the body. On the one hand, gene delivery systems can regulate the expression of relevant proteins; on the other hand, nanosystems can act as functional components, integrating organically with cells to perform specialized functions.

#### 3.1.1 Nanotechnology-assisted gene regulation in cells

Cellular gene expression refers to the process by which cells utilize their genetic information to synthesize proteins. This process involves three main steps: DNA transcription to mRNA, mRNA processing, and translation [103]. Therefore, delivering CRISPR-Cas9 systems for gene editing, directly delivering mRNA into target cells, or delivering siRNA to regulate mRNA expression are currently the main strategies for gene regulation [103,104]. However, gene therapy faces some challenges and obstacles in vivo. Single DNA or mRNA molecules lack specificity and are susceptible to degradation. Additionally, they are restricted by factors such as the immune system, cell membrane barriers, and local tissue environments, leading to low gene delivery efficiency [105]. Moreover, introducing exogenous genes into the body may trigger immune reactions, including cell-mediated and humoral immune responses [106].

To address these issues, nanosystems are frequently employed as carriers for gene delivery, allowing for precise control over the gene delivery process. Through the rational design of parameters such as size, shape, surface properties, and cargo release kinetics, nanocarriers can improve biocompatibility and protect DNA or RNA molecules from degradation, thus enhancing gene stability and bioavailability. Additionally, nanocarriers can deliver genetic payloads to specific cell types or organs through targeting strategies, improving the effectiveness of gene delivery. For example, nanosystems delivering the CRISPR/Cas9 DNA editing system can specifically regulate intestinal macrophages, offering a treatment for inflammatory bowel disease. Since mRNA can be directly translated into proteins, it enables rapid and temporary gene expression, making mRNA delivery a research hotspot. Studies have demonstrated that mannose-modified lipid nanoparticles (STLNPs-Man) can target dendritic cells (DCs), promoting antigen protein expression within DCs and enhancing antigen presentation, showing significant anti-tumor potential [107].

Similarly, siRNA delivery allows binding to and degradation of target gene mRNA, leading to specific inhibition of gene expression. siRNA typically provides temporary effects without causing long-term genetic alterations, which contributes to higher biosafety. Yang and colleagues developed a nano-delivery platform for lncBCMA siRNA, showing effective treatment of triple-negative breast cancer [108].

Through the intrinsic responsiveness to internal environments, such as lysosomal escape, nanosystems can release gene-editing tools, although this approach presents the risk of uncontrollable gene switches. To address this issue, researchers have combined external stimuli such as heat, light, sound, and magnetism, using nanoparticles as carriers for external signal transduction, to convert external signals into input signals recognizable by gene switches, thereby achieving spatial and temporal control of gene expression. For example, Ortner et al. encapsulated magnetic nanoparticles and cells containing inducible heat shock promoter elements into implantable capsules, applying an external alternating magnetic field to generate local heat within the capsules, inducing the expression of a luciferase reporter gene [109]. Wang et al. accomplished this by using photothermal-responsive conjugated polymer nanoparticles to convert near-infrared light into heat energy. The heat-inducible heat shock protein-70 promoter initiates transcription of the downstream EGFP gene upon heat shock, leading to the production of green fluorescent protein within living cells. This approach facilitates precise and timely remote control of gene expression with high spatial and temporal resolution within cells [110].

#### 3.1.2 Nanosystems as specialized functional components in cells

**Fig. 5:**
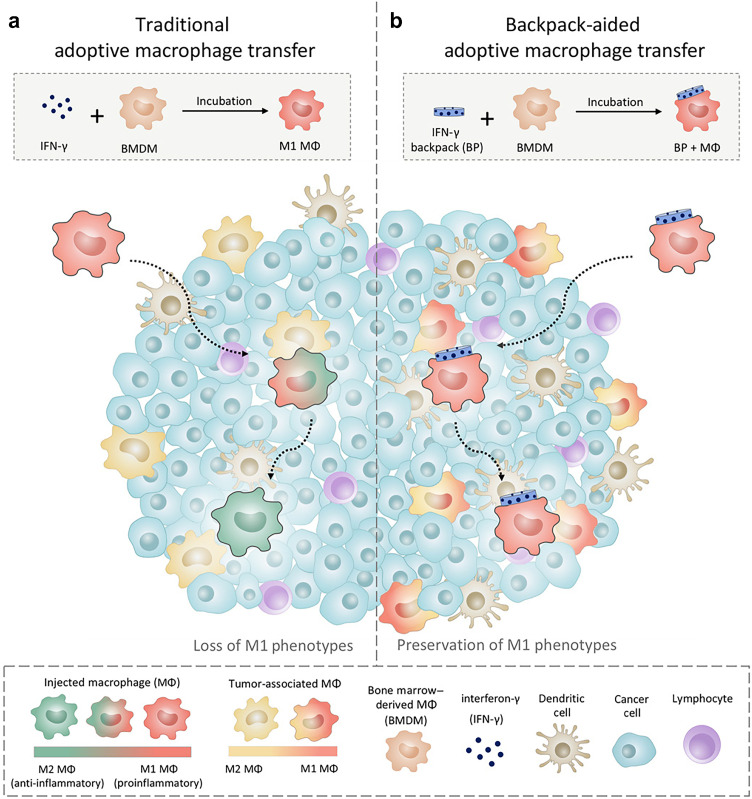
**Illustration of cellular 'backpacks' engineered to sustain proinflammatory states in adoptively transferred macrophages (MΦ) for therapeutic purposes.** (a) Ex vivo-polarized MΦs, treated with IFN-γ, often revert to anti-inflammatory phenotypes upon entering solid tumors. (b) Macrophages equipped with IFN-γ–loaded cellular backpacks, however, retain their proinflammatory phenotypes within the tumor microenvironment, exerting influence on local TAMs. This approach enables backpack-carrying macrophages to maintain their activation states even within the immunosuppressive environment of solid tumors, supporting a sustained and potent antitumor immune response [111]. Reprinted with permission.

Nanosystems can adhere to the cell surface through surface modifications, functioning as "cell backpacks" that continuously secrete relevant cytokines to regulate cellular activity. For example, Samir Mitragotri and colleagues designed disc-shaped "backpacks" using microcontact printing technology, consisting of two layers of biocompatible polymers, poly(lactic-co-glycolic acid) (PLGA), sandwiching hydrophilic polyvinyl alcohol (PVA) and cytokines like interferon-gamma (IFNγ) (Fig. 5) [111]. These backpacks adhered to macrophages and sustained IFNγ release for at least 60 h, promoting the transition of macrophages to the M1 phenotype, thereby inhibiting tumor growth and reducing metastasis. Similarly, studies have shown that protein nanogels (NGs) can be used to load large amounts of protein drugs on T cells. These NGs selectively release the cargo to promote T-cell receptor activation, enhancing their therapeutic efficacy. Similarly, by attaching disc-shaped microparticles called “backpacks” to neutrophils, they can keep the cells in an anti-tumor (N1) state [112].

Nanosystems can also act as nanozymes, mimicking natural enzymes like superoxide dismutase (SOD), catalase (CAT), and peroxidase (POD) within the cellular microenvironment [113]. These nanozymes exhibit great potential for regulating cellular metabolism. For instance, nanozymes with SOD-like and CAT-like activities catalyze the conversion of superoxide radicals (O₂⁻), reducing intracellular ROS levels [114]. This modulation can influence signaling pathways and metabolic states, particularly in pathways related to oxidative stress. Additionally, nanozymes often exhibit stable activity in extreme conditions, such as high temperatures or pH, expanding their potential applications compared to natural enzymes [113].

Nanosystems, functioning as cellular biosensors, exhibit significant potential in cell monitoring and detection, especially for real-time assessment of cellular metabolism, signal transduction, and environmental changes. These nanosensors can operate either inside cells or on their surface to detect and respond to various biomolecular or physical alterations [115]. For example, nanoparticles such as quantum dots and gold nanoparticles can be engineered as fluorescent sensors to monitor specific ions (e.g., Ca²⁺, Na⁺) or molecules (e.g., glucose, ROS) [116,117]. When the target molecules interact with these sensors, the fluorescence intensity or wavelength changes, providing a real-time method to monitor intracellular biological events. Additionally, electrochemical sensors based on nanomaterials like carbon nanotubes or graphene can detect cellular metabolites, redox states, and other biochemical signals [118,119]. These sensors sense changes in the extracellular fluid's chemical composition and record current fluctuations, offering high sensitivity for monitoring complex biological processes within cells. Such capabilities are valuable for studying intracellular dynamics and metabolic pathways in detail.

In conclusion, traditional synthetic biology can be likened to a production line where alterations to the blueprint (DNA) lead to the synthesis of desired products (proteins). However, this process is often limited by the complexity of in vitro synthesis. Advances in nanotechnology have enabled precise regulation of each phase of biosynthesis within the body. Moreover, nanosystems can function as final products, bypassing complex biological synthesis steps and rapidly exerting biological effects. This accelerates therapeutic efficacy and achieves the intended biological outcomes.

### 3.2 Assembling systems of nanomaterials and bacteria

Bacteria exert various influences on human beings, encompassing both beneficial and harmful effects. The microbiota in the human digestive tract aid in food digestion, nutrient absorption, and synthesis of essential vitamins such as vitamin K and B-complex vitamins, vital for human health [120]. Additionally, certain bacteria possess biodegradative capabilities, facilitating the decomposition of organic matter in the environment and aiding in soil and water purification [121]. Interestingly, bacteria find widespread applications in industrial processes such as biopharmaceutical fermentation, food processing, and environmental engineering [122]. However, pathogenic bacteria not only cause infectious diseases such as bacterial food poisoning, pneumonia, and diarrhea but may also disrupt the immune system, leading to allergic reactions or autoimmune diseases [123]. Therefore, the impact of bacteria on humans is complex and diverse. Utilizing nanotechnology to modify bacteria or construct nano-bacteria assembling systems can achieve a wider range of intriguing and useful functions.

Engineered bacteria through NSB refer to the modification and control of bacteria using nanotechnology to achieve specific functionalities or applications [124]. These engineered bacteria, often termed as nano-bioengineered bacteria, find applications across various fields including medicine, environment, energy, and more [125]. They can be designed as delivery carriers for precise transport of drugs to targeted cells or tissues, or for utilization in gene therapy and cancer treatment applications [109]. Through NSB, we can build nano-bacterial assembling systems to meet the requirements of specific applications. The nano-bacteria assembling system is a hybrid system that integrates nanotechnology with bacterial applications. Typically, this system exploits the properties of nanomaterials and the biological activity of bacteria to achieve specific functions. In this system, nanomaterials can serve as carriers, loading drugs or other biologically active substances on their surface or internally, while bacteria can act as transporters, assisting in the delivery of these substances to specific locations or tissues. The configuration of these assembling systems can be flexibly adjusted and meticulously optimized to meet the precise requirements of various applications, thereby striving for superior functional performance with heightened efficiency.

**Fig. 6:**
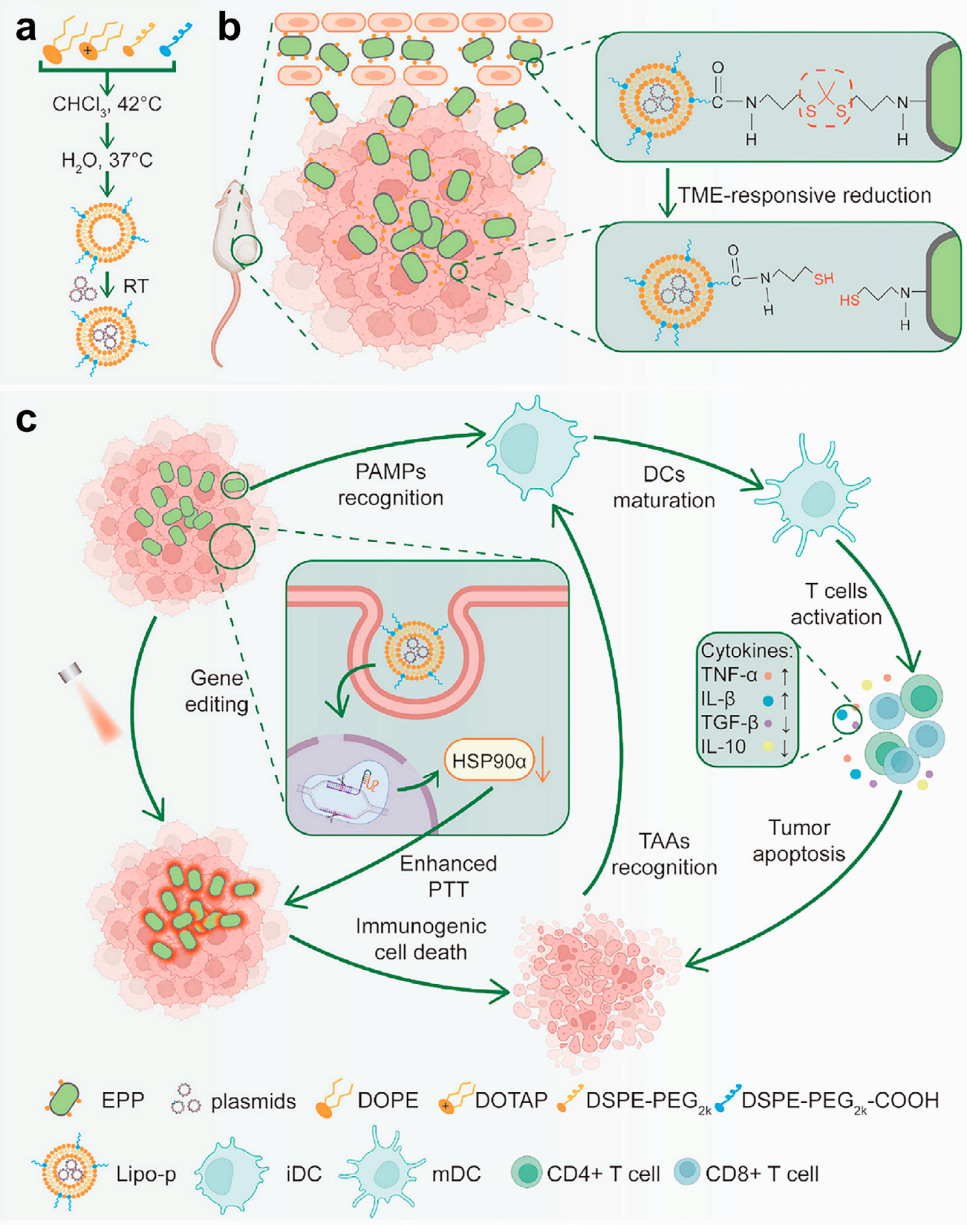
**Design of the EcN-assisted CRISPR-Cas9 system delivery and photothermal-sensitized immunotherapy in the deep tumor.** Nonpathogenic EcN was modified with a polydopamine coating, forming EP, which provides both biocompatibility and photothermal capabilities. Cationic carboxylated liposomes containing CRISPR/Cas plasmids (Lipo-P) for *Hsp90α* knockout were linked to EP via ROS-responsive linkers, resulting in EPP. Upon EcN localization to hypoxic tumor areas, Lipo-P is released in the TME, reducing tumor cell heat resistance. Mild photothermal therapy (mPTT) with near-infrared light (808 nm) enhances the system's effects, triggering immunogenic cell death, which activates antitumor immunity and converts 'cold' tumors to 'hot.' This CRISPR-aided approach showed promising results in vitro and in vivo for deep tumor immunotherapy. (a) Schematic diagram of Lipo-P. (b) Hypoxia-specific delivery of CRIPSR-Cas9 system using ROS-responsive bonds via bacteria. (c) Probiotics-based biotherapy of CRISPR-assisted mPTT and enhanced immunotherapy [126]. Reprinted with permission.

Because of the hypoxic conditions and immune suppression prevailing within tumor tissues, bacteria exhibit promising potential as targeted carriers for drug delivery [110]. Luo et al. have introduced a novel photothermal-sensitive immunotherapy approach, utilizing dopamine-coated *E. coli* Nissle 1917 (EcN) as carriers for liposomes containing CRISPR-Cas9 plasmids (Lipo-P) (Fig. 6) [126]. Upon targeted accumulation in hypoxic tumor cores, EcN releases the Lipo-P liposomes in response to ROS, thereby diminishing the thermal resistance of cancer cells via depletion of Hsp90α. When subjected to near-infrared irradiation, dopamine generates sufficient heat for effective tumor ablation. Additionally, the mild photothermal therapy induces immunogenic cell death, while the concurrent bacterial infection within the tumor core triggers innate immunity, leading to a robust immune response. This bacterial-assisted strategy shows potential for treating deeply-seated tumors. Additionally, Zheng et al. orchestrated *E. coli* carrying NO synthase, facilitating the transportation of carbon nitride (C_3_N_4_), employing a photothermal bacterial metabolite therapy for breast cancer treatment [127]. Leveraging the photoconversion capability of C_3_N_4_, modified CCN@E. coli metabolizes NO_3_^–^ to produce antineoplastic NO under photo-irradiation, ultimately inhibiting approximately 80% of tumor growth.

Nevertheless, the utilization of live bacteria in vivo raises potential biosafety concerns. Inactivated bacteria, while reducing toxicity, still maintain immunogenicity on their surface proteins and polysaccharides, leading to rapid recognition and engulfment by immune cells. Capitalizing on this aspect, Li et al. utilized inactivated bacteria as carriers, with the surface loaded with bimetallic gold-platinum nanozymes [128]. It was observed that following intravenous injection, inactivated bacteria could accumulate at tumor sites through a ‘hitchhiking mechanism’ with CD11b^+^ cells. Under low-dose X-ray irradiation, this nano-bacterial combination system induced a robust tumor inflammatory response, significantly alleviating tumor hypoxia and immunosuppression, thereby inhibiting tumor growth and metastasis.

In summary, with the continuous advancement of technology and in-depth exploration of its potential applications, nano-bacteria assembling systems still hold immense development potential and may emerge as significant tools in the fields of biomedicine and bioengineering in the future. Nevertheless, a comprehensive assessment of their potential toxicity and biocompatibility is imperative. Furthermore, ensuring sufficient specificity of the system towards target cells or tissues poses a challenge to avoid unnecessary side effects and toxic reactions.

### 3.3 Assembling systems of nanomaterials and viruses

Viruses are microscopic infectious agents composed of a protein coat enclosing a nucleic acid core (either DNA or RNA). Their size typically ranges from 20 to 300 nanometers and they can only be observed under an electron microscope [129]. Viruses lack the machinery for independent biological activity and rely on infecting host cells to replicate and reproduce [130]. The viral lifecycle typically involves stages such as attachment, entry, replication, assembly, and release [131]. Upon infecting a host cell, the virus releases its genetic material into the host cell, hijacking the host cell's machinery for replication, and ultimately releasing newly formed viral particles. Additionally, viruses often exhibit specific host cell tropism, meaning they can only infect particular types of cells, including animal viruses, plant viruses, or bacterial viruses.

Therefore, based on their unique infection mechanisms, viruses possess inherent attributes of high specificity, efficiency, and programmable genomes, rendering some low-pathogenic viruses or virus-like particles ideal carriers in synthetic biology, facilitating the delivery of target genes into cells [132]. Adeno-associated viruses (AAVs) and lentiviruses have garnered extensive usage in gene therapy, attributed to their low immunogenicity and capability for site-specific integration [133,134]. Among them, AAVs have achieved significant success in gene therapy, with FDA-approved AAV-based therapies such as Luxturna by Spark Therapeutics and Zolgensma by Avexis, used for treating inherited retinal diseases and spinal muscular atrophy, respectively [135,136].

Previous studies have frequently utilized viral vectors for gene therapy delivery, with AAV being particularly prominent due to its efficient ability to traverse species barriers for cell infection and its low immunogenicity, thus reducing the risk of inflammatory reactions [137]. However, compared to conventional gene therapies, the CRISPR/Cas9 system is significantly larger, exceeding the maximum packaging capacity of AAV vectors by 4.7 kb [138]. Thus, modifications to the AAV vector are required, such as utilizing smaller Cas9 variants like SaCas9 or splitting the delivery system into two vectors [139]. Incorporating the coding sequence of the smaller Cas9 ortholog SaCas9 into an expression cassette allows for the inclusion of effector coding sequences as epigenetic modulators, thereby enhancing Cas9 regulatory activity while keeping plasmid size within the carrying capacity of AAV. For instance, Charis et al. established a CRISPRi system using dSaCas9, successfully suppressing DUX4 mRNA expression in vitro, mitigating facioscapulohumeral muscular dystrophy [140]. Dual AAV vectors comprise separately engineered plasmids encoding Cas9 and sgRNA. Although transfection into cells yields complete Cas9 protein and sgRNA for gene regulation, this approach carries a higher risk of off-target effects. Additionally, Wen et al. illustrated efficient reduction of mutant hTTR expression within a transgenic mouse model of transthyretin amyloidosis by employing single AAV-mediated CRISPR-Nme2Cas9 [141].

Besides AAV, lentiviruses are also utilized for nucleic acid delivery in biosynthetic engineering [142]. Lentiviruses, retroviruses capable of infecting both dividing and non-dividing cells, are frequently employed as delivery vectors owing to their enhanced payload capacity [143]. The entire CRISPR/Cas9 system can be accommodated within lentiviral vectors. Nonetheless, because of the random integration of lentiviral genes into the host genome, they often elicit immune reactions and may even lead to cancer. To address off-target effects and immune responses more effectively, lentiviral vectors often pre-load Cas9 protein, ensuring a safer approach to genome editing [144]. Lin et al. employed single-cell cloning and lentiviral transduction techniques to obtain the CRISPR/Cas9 system and facilitate the knockout of the target gene [145]. Michael et al. reported a novel approach for inserting and stably expressing large genetic payloads, alongside a method for simultaneously inserting two genes into two endogenous loci [146]. They employed a lentivirus with an integration-defective enzyme encoding payload flanked by homology arms and ‘cutting sites’, capable of inserting the payload upstream and within the frame of endogenous essential genes, followed by electroporation-mediated delivery of CRISPR-associated RNP complexes. This gene editing system showed remarkable proficiency in inserting and maintaining the stable expression of large payloads, including two challenging-to-express viral antigens, within primary T cells, all while exhibiting minimal cytotoxicity.

Similar to cell membranes and bacterial membranes, virus-like nanoparticles (VLPs) derived from viral capsids serve as excellent nanocarriers for delivery, mimicking the behavior of viral infections to transport relevant genes and drugs [19]. Their surfaces typically display characteristics of viral surface proteins, which can bind to host cells. Because they lack genetic material (DNA or RNA), they are incapable of infecting host cells, thereby ensuring enhanced biosafety. Rather than infecting host cells, efficient nucleic acid delivery can be achieved by loading cargo inside, such as CRISPR systems or mRNA. The CRISPR/Cas9 system has been widely applied for gene editing in human cells, where transient expression of Cas9 protein can alleviate off-target effects. Lu et al. developed lentivirus-like bio-nanoparticles (LVLPs) capable of carrying up to 100 copies of Staphylococcus aureus Cas9 (SaCas9) mRNA [147]. In the presence of guide RNA, SaCas9 LVLPs efficiently edited the genome both in vitro and in vivo while mediating temporary SaCas9 expression.

**Fig. 7:**
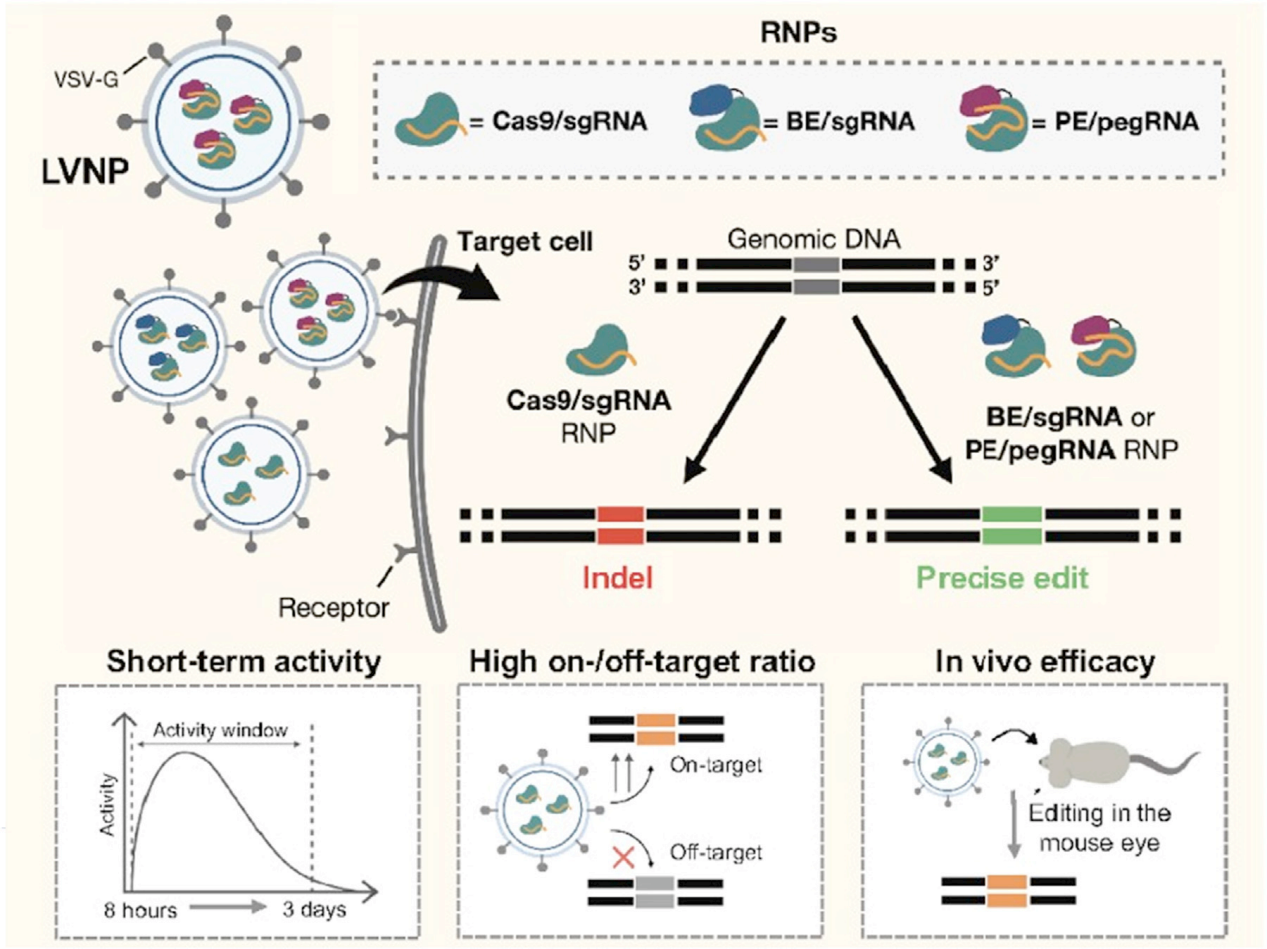
**By engineering lentivirus-derived nanoparticles (LVNPs) to facilitate RNP delivery, the authors demonstrate effective administration of SpCas9 as well as SpCas9-derived base and prime editors (BE/PE) leading to gene editing in recipient cells.** Unique Gag/GagPol protein fusion strategies facilitate RNP packaging in LVNPs, and refinement of LVNP stoichiometry supports optimized LVNP yield and incorporation of therapeutic payload. They demonstrate near instantaneous target DNA cleavage and complete RNP turnover within 4 days. As a result, LVNPs provide high on-target DNA cleavage and lower levels of off-target cleavage activity compared to standard RNP nucleofection in cultured cells. LVNPs accommodate BE/sgRNA and PE/epegRNA RNPs leading to base editing with reduced bystander editing and prime editing without detectable indel formation. Notably, in the mouse eye, they provide the first proof-of-concept for LVNP-directed in vivo gene disruption [152]. PE, prime editor; BE, base editor; VSV-G, vesicular stomatitis virus glycoprotein; pegRNA, prime editing guide RNA. Reprinted with permission.

However, the majority of people's body fluids already contain antibodies against AAV, which restricts the use of AAV for systemic gene delivery. When naturally occurring, viruses do not meet researchers’ needs, genetic engineering can be used to reduce viral pathogenicity or modify the surface proteins of virus capsids, creating virus-like shells with specific functions. These modified shells can carry specific nano ‘cargo’, thereby improving targeted delivery, immune activation, or controlled release. For instance, to overcome the limitations of natural AAV, surface modifications of AAV are performed through capsid engineering, where the CAP genes undergo modifications to incorporate a novel peptide or tag (creating a chimeric capsid), fuse two capsids together (forming a mosaic capsid), or package an AAV gene construct from one serotype into a different capsid serotype (transcapsidation) [[148], [149], [150]]. Recently, Williams et al. engineered AAV to accomplish targeted gene knock- in mouse T cells, demonstrating high transduction efficiency [151]. This system holds potential for non-nuclear transfection of DNA, CRISPR-Cas9-mediated knockout, and targeted integration of large transgenes in experimental T cell immunology, paving the way for new avenues. It is widely recognized that compared to traditional viral and non-viral gene and RNA delivery methods, delivering RNP complexes composed of Cas protein and sgRNA provides the benefits of transient editing activity and enhanced safety. Jakob et al. developed RNP packaging by designing lentivirus-derived nanoparticles (LVNPs) using a Gag/GagPol protein fusion strategy, thus promoting RNP encapsulation in LVNPs (Fig. 7) [152]. Furthermore, for in vivo gene therapy to be successful, precise gene delivery to therapeutically relevant cells is critical. Samuel et al. designed engineered AAVs vectors displaying engineered CD4 and CD32a bispecific anchoring repeat proteins on the virus surface for targeting HIV reservoir T cells [153]. Cryo-electron tomography showed that the engineered capsid structure remained unchanged. Surprisingly, the bispecific AAV transduced CD4/CD32a double-positive cells much more efficiently than single-positive cells. This bispecific antibody targeting provides a new option for preparing novel vectors to improve the specificity and safety of in vivo gene therapy. As multifunctional carriers, superparamagnetic iron oxide nanoparticles have been designed to increase the effectiveness of gene delivery. To improve the delivery of genes to HEK293T and PC12 cell lines, Huang et al. created a magnetically guided AAV delivery methodology [154]. Wild-type AAV2 and a novel AAV vector, AAVr3.45, were employed as gene carriers, with AAVr3.45 evolved directly from previous studies to possess multiple cell tropisms. Moreover, the inherent affinity of each viral vector for heparin was leveraged to immobilize the virus onto heparin-coated SPIONs. Magnetic-guided AAV delivery led to rapid and efficient cell transduction. Furthermore, compared to non-magnetic or non-guided systems, the magnetic-guided encoding of nerve growth factor (NGF) AAV delivery system produced sufficient functional NGF, leading to a strong neurite outgrowth rate in PC12 cells. The successful establishment of the magnetic-guided AAV delivery system enables efficient and rapid infection of target cells, providing a sturdy foundation for a wide array of gene therapy application. Moreover, Kim et al. genetically modified AAV to express six histidine residues on the capsid [155]. The histidine residues interacted with modified superparamagnetic iron oxide nanoparticles, resulting in magnetically driven virus-mimetic nanoparticles. In the presence of a magnetic field, this delivery system remarkably amplifies cell transduction efficiency, concurrently reducing viral transduction time. Besides genetic engineering modifications, combining non-viral and viral vectors is also a feasible approach.

In conclusion, nano-virus assembling systems hold immense promise in NSB, spanning applications such as nanoparticle assembly, gene delivery, and therapy. Through engineering modifications and functionalization of viruses, scientists can develop novel and highly efficient viral vector applications, leading to significant advancements in the fields of biomedicine and nanotechnology.

## 4 Assembling systems in different biomedical application

### 4.1 Cancer therapy

In the past, endeavors have focused on harnessing targeted and efficacious drugs for cancer therapy. However, traditional chemotherapy drugs have limited targeting ability, poor controllability, and often result in multiple side effects [156]. the utilization of nanobiotechnology facilitates the design and fabrication of precise nanodrug delivery systems These carriers can achieve specific recognition and binding to tumor cells through surface functionalization, thereby enhancing targeting and therapeutic effects of drugs while minimizing toxicity to normal tissues [157]. These targeted drug delivery systems have the capability to transport therapeutic agents such as drugs, genes, and RNA. NSB not only enables the integration of various functional nanomaterials and biomolecules but also facilitates the construction of multifunctional nanodrug systems. These systems can serve various functions including diagnosis, treatment monitoring, photothermal therapy, immunotherapy, and other functions, thus contributing to comprehensive cancer treatment outcomes.

Nanotechnology facilitates the preparation of metal nanoparticles with photothermal conversion properties, which find applications in photothermal therapy and photodynamic therapy. These nanoparticles generate localized heat or ROS upon light stimulation, leading to thermal or oxidative damage to tumor cells, thereby achieving cancer treatment. Multi-component deoxyribonucleic acid enzymes (MNAzymes) hold immense potential in gene therapy, yet their ability to target diseased tissues and further accomplish synergistic gene regulation remains largely unexplored. Yan et al. encapsulated calcium efflux inhibitor curcumin and hydrophobic photothermal dye IR780 within DSPE-PEG-RGD micelles [158]. The MNAzyme was incorporated into the hydrophilic PEG layer and subsequently encapsulated with calcium phosphate via biomineralization Upon targeting tumors via RGD, the nanoparticles induced internalization and released the payload in an acidic environment. Curcumin maintained elevated intracellular Ca2^+^ levels, which, as a cofactor, aided MNAzyme in depleting tumor cells’ overexpressed miRNA-21 by consuming Ca^2+^. This action facilitated self-assembly and mediated the cleavage of heat shock protein mRNA. Silencing miRNA-21 enhanced PTEN gene expression, rendering tumor cells sensitized to photothermal therapy. Under laser exposure, IR780-mediated photothermal effect was significantly amplified. In vivo experiments demonstrated that this nanosystem effectively inhibited the growth of pancreatic cancer in mice.

**Fig. 8:**
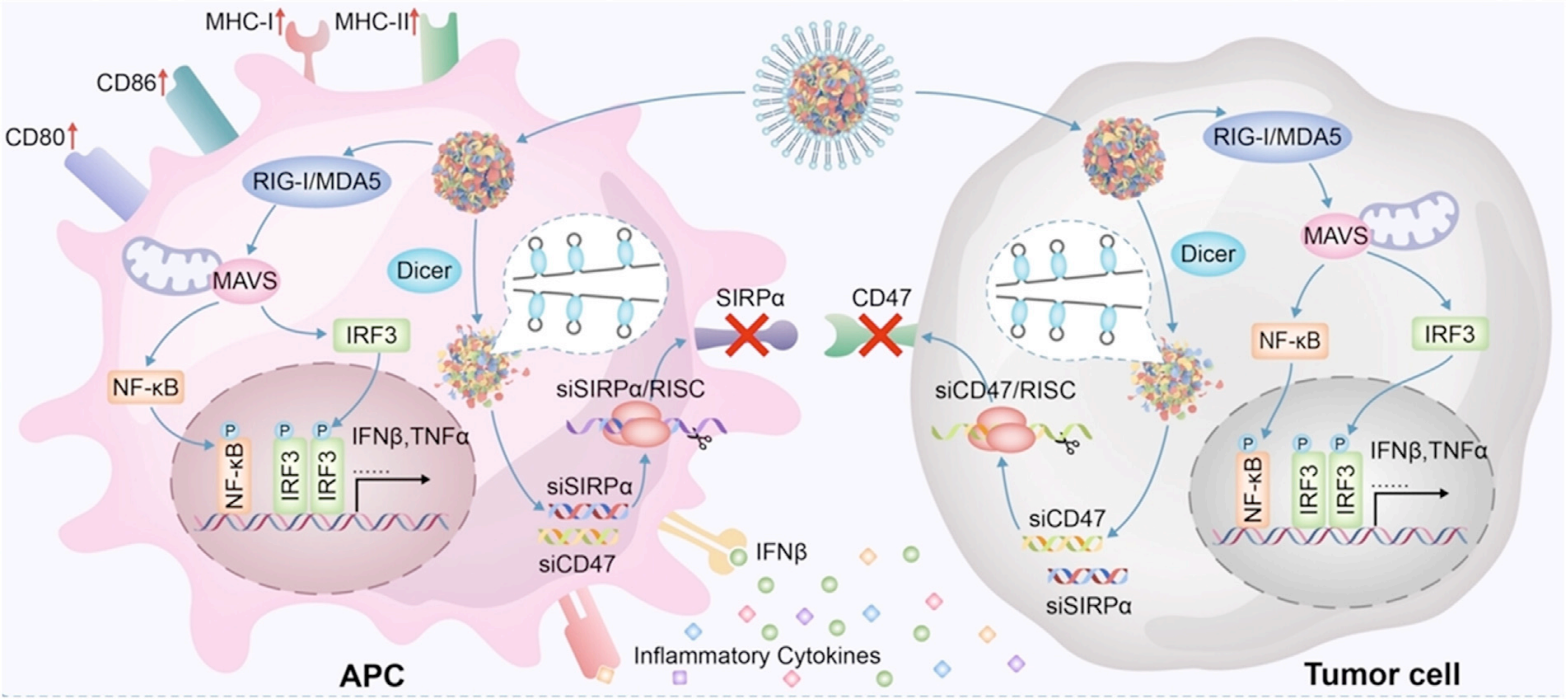
**A nanoadjuvant is constructed from long RNA strands generated by rolling circle transcription (RCT), which were further modified with cationic liposomes.** Due to the densely packed RNA, this nanoadjuvant efficiently activates RIG-I/MDA5 signaling within APCs, inducing dendritic cell maturation and promoting TAMs to shift toward an anti-tumor M1-like phenotype. By designing a specific template, the RCT-generated RNA includes siRNA sequences that target CD47 in tumor cells and SIRPα in APCs, effectively blocking the CD47-SIRPα checkpoint. This dual-targeted inhibition enhances APC phagocytosis and subsequently supports the cross-priming of T cells, stimulating anti-tumor immunity. The study offers a streamlined, effective RNA-based nanoadjuvant approach for cancer immunotherapy [162]. Reprinted with permission.

NSB enables the design and synthesis of immunomodulatory nanomaterials, which serve to improve the efficacy of immunotherapy [159]. These nanomaterials can stimulate the host immune system, facilitating the immune recognition and clearance of tumor cells. Notably, antigen-presenting cells (APCs) play a pivotal role in anti-tumor immunity, as they are responsible for capturing, processing, and presenting tumor antigens to T cells [160]. However, their activation is often constrained by the lack of adjuvants and inhibition from immune checkpoints, such as CD47-SIRPα [161]. Therefore, Xu et al. employed cationic liposomes to deliver long RNA nanoparticles produced via rolling circle transcription as a novel nano adjuvant. Capitalizing on the high payload capacity of RNA, this nano adjuvant effectively activates the RIG-I/MDA5 signaling pathway, leading to dendritic cell maturation and polarization of TAMs toward M1 phenotype (Fig. 8) [162]. Moreover, the two different siRNAs that target SIRPα in APCs and CD47 in tumor cells are present in the RNA nanoparticles, thereby blocking the CD47-SIRPα checkpoint. The potent stimulation of the RIG-I/MDA5 signaling pathway coupled with dual-gene silencing targeting the CD47-SIRPα checkpoint synergistically augments APC phagocytic activity. This enhances the priming of effector T cells and promotes the activation of anti-tumor immune responses. Together, these findings highlight a novel and efficient RNA nano adjuvant for cancer immunotherapy. In summary, NSB holds tremendous potential in the field of cancer therapy, offering novel treatment strategies and approaches that can provide patients with more effective and safer therapeutic options.

### 4.2 Autoimmune diseases therapy

Differing from tumor immunity, autoimmune diseases represent a category of disorders wherein the immune system erroneously targets the body's own tissues and organs, encompassing type 1 diabetes (T1D), multiple sclerosis, rheumatoid arthritis, systemic lupus erythematosus, among others [163]. NSB can be harnessed for designing and fabricating drug delivery systems endowed with specific targeting and immune-modulating functionalities, facilitating the delivery of therapeutic nucleic acids, antigens, or drugs to specific target cells or APCs, thereby inducing tolerance to self-antigens. This approach holds the potential to dampen excessive immune responses, alleviate disease symptoms, control disease progression, and concurrently mitigate damage to healthy tissues [164].

T1D, a complex interplay of genetic, environmental, and other unknown factors, results in the immune system erroneously targeting the pancreatic islet β-cells [165]. This leads to reduced or complete cessation of insulin secretion, leading to elevated blood glucose levels and the manifestation of diabetic symptoms. Besides insulin replacement therapy, researchers are dedicated to inducing immune tolerance to fundamentally cure the disease [166]. Wilhelm et al. identified that transfection reagents based on liposomes and polyethyleneimine (IVF2 and JetPEI, respectively) serve as tools for precisely delivering siRNA to pancreatic APCs in vivo [167]. They observed that early administration of liposomal/Alox15-specific siRNA complexes confer prolonged protection against T1D in non-obese diabetic mice. Downregulation of Alox15 in pancreatic CD11b^+^ cells significantly upregulate various co-stimulatory molecules (particularly the PD-L1 pathway), while the increase in regulatory T cells in the pancreas favors the induction of immune tolerance. In summary, siRNA-based therapies for T1D hold promising applications.

Multiple sclerosis is an autoimmune disease characterized by inflammatory demyelination of the central nervous system, primarily mediated by activated CD4^+^ myelin-reactive T cells [168]. A key challenge is effectively delivering specific antigens to APCs in a non-inflammatory background to induce antigen-specific immune tolerance. Christina et al. discovered that messenger RNA modified with 1-methylpseudouridine (m1Ψ mRNA) can diminish the inflammatory effects of mRNA stimulation in vivo [169]. They utilized liposomes loaded with m1Ψ mRNA encoding specific antigens to deliver antigens to splenic CD11c^+^ APCs in the absence of co-stimulatory signals. This leads to a decrease in effector T cells and an increase in regulatory T cells, thereby inducing immune tolerance Additionally, these regulatory T cells exert potent bystander immune suppression, thereby ameliorating multiple sclerosis induced by both homologous and heterologous self-antigens.

Rheumatoid arthritis is also an autoimmune chronic systemic inflammatory disease characterized by inflammation of synovial tissues. Aberrantly activated T cells, synovial fibroblasts, and macrophages produce inflammatory cytokines such as IL-1, IL-6, IL-17, and TNF-α [170]. Studies have shown that reducing TNF-α at the lesion site contributes to disease treatment [171]. Abdulaziz et al. presented an acid-sensitive detachable polyethylene glycol-modified solid lipid nanoparticle formulation of TNF-α-siRNA, known as AS-TNF-α-siRNA-SLNs. This formulation involved integrating lipid-hybridized TNF-α-siRNA into solid lipid nanoparticles comprised of biocompatible lipids like phospholipids and cholesterol [172]. It exhibited a high encapsulation rate (> 90%) and release rate (> 5%) of siRNA. In a model of rheumatoid arthritis resistant to methotrexate treatment, AS-TNF-α-siRNA-SLNs notably decreased TNF-α levels at the lesion site, paw thickness, bone loss, and tissue histopathological scores. This novel siRNA nanoparticle formulation, which regulates the biosynthetic process, holds promise for the effective treatment of arthritis. In conclusion, nanobiotechnology shows tremendous potential in the treatment and diagnosis of autoimmune diseases. By precisely controlling and designing the interaction between nanomaterials and biological systems, more effective and safer treatment methods can be developed, thereby providing better medical care for patients.

### 4.3 Biocatalysis

The utilization of NSB in biocatalysis is of notable significance. Nanotechnology facilitates the creation of biomimetic nanozymes possessing specific structures and functions reminiscent of organelles [173]. These biomimetic nanozymes, through surface functionalization, can achieve substrate-specific recognition and efficient catalytic conversion, thereby regulating cellular metabolic synthesis processes. In contrast to conventional chemical catalysts, nanobiocatalysts exhibit higher catalytic activity and improved selectivity [174,175]. Nanobiotechnology also allows for microenvironment-responsive functionality, such as responsiveness to factors like H_2_O_2_ concentration, temperature, pH, solvent, etc., enabling specific metabolic regulation and reducing off-target effects and biological toxicity [176].

**Fig. 9:**
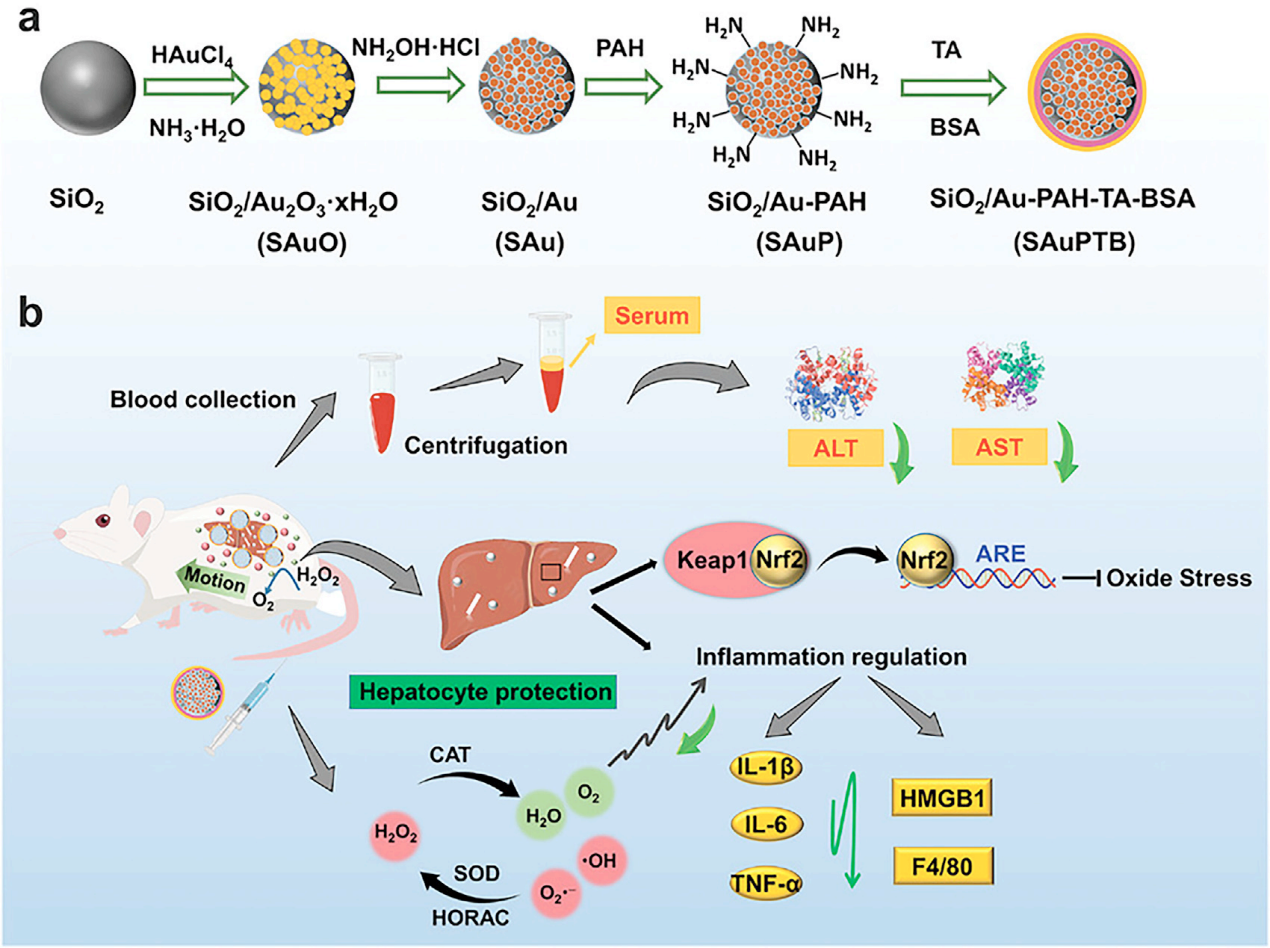
**A self-propelled nanozyme (SAuPTB) has been developed, featuring ultrasmall gold nanoparticles on a silica-tannic acid hybrid structure, capable of mitigating acetaminophen-induced liver damage.** By efficiently scavenging ROS and modulating inflammatory responses, SAuPTB showcases multiple enzyme-like activities and enhanced movement in the presence of hydrogen peroxide (H_2_O_2_). In vitro studies demonstrate that SAuPTB effectively reduces ROS levels, promotes cell viability in H_2_O_2_-stimulated models, and lessens cytotoxic effects in APAP/H_2_O_2_-treated AML12 cells [179]. (a) Preparation of SAuPTB nanoparticles. (b) How SAuPTB relieves drug-induced liver injury by attenuating ROS and regulating inflammation. TA: tannic acid; BSA: bovine serum albumin. PAH: poly (allylamine hydrochloride); ALT: Alanine aminotransferase; AST: Aspartate aminotransferase. Reprinted with permission.

The application of nanozymes to regulate organismal metabolic processes for disease treatment represents a significant utilization of biocatalysis. For instance, periodontitis, a chronic inflammatory disease originating from dental plaque, is characterized by the excessive accumulation of ROS, matrix metalloproteinases (MMPs), and other substances, leading to periodontal tissue destruction [177]. Xu et al. proposed a multifunctional nanoenzyme on-demand release platform (TM/BHT/CuTA) composed of copper-based nanozymes (copper tannate-coordinated nanosheets, CuTA NSs) and a glyceryl monooleate/2,6-di‑tert‑butyl‑4-methylphenol (TM/BHT) hydrogel self-assembled into TM/BHT/CuTA hydrogel system [178]. Electrostatic adsorption enables effective retention of the negatively charged hydrogel at positively charged inflammatory sites. At the inflammatory site, MMPs facilitate the degradation of the hydrogel, thereby releasing CuTA nanozymes. These nanozymes mimic the cascade process of superoxide dismutase and catalase, effectively clearing excessive ROS and exerting anti-inflammatory effects. Additionally, CuTA nanozymes can promote macrophage polarization from M1 to M2 phenotype via the Nrf2/NF-κB pathway, alleviating inflammation, and also enhance the expression of osteogenic genes, accelerating tissue regeneration in periodontitis. This multifunctional nanoenzyme on-demand release therapy provides a new strategy for treating periodontitis. Meanwhile, Zhou et al. devised a self-propelled silica-supported ultrasmall AuNPs-tannic acid hybrid nanozyme (SAuPTB), demonstrating its efficacy in mitigating acetaminophen-induced liver injury. This nanozyme effectively scavenges excess ROS and regulates inflammation (Fig. 9) [179]. In vivo investigations reveal that SAuPTB concentrates at inflammatory sites in liver, catalyzing the reduction of ROS levels in hepatic cells and ameliorating liver damage. Furthermore, SAuPTB activates the nuclear erythroid 2-related factor 2 (Nrf2) pathway, leading to the upregulation of antioxidant genes that mitigate oxidative stress, thereby exhibiting anti-inflammatory properties.

Biocatalytic interventions not only restore the organism to homeostasis by regulating abnormal metabolism but also augment the organism's defense and recovery capabilities by promoting catalytic activity. For instance, Wu et al. synthesized a biomimetic nanozyme catalyst (Bi-PCN-222) with rapid and efficient antibacterial and wound healing properties. Based on Schottky interface-driven charge transfer, Bi-PCN-222 MOF possesses both oxidase-like and peroxidase-like activities [180]. As a result, a substantial quantity of free electrons can be captured, leading to the generation of ROS. When Staphylococcus aureus comes into contact with Bi-PCN-222, the redox potential can penetrate the bacterial membrane and disrupt respiration and metabolism, thereby killing the bacteria. Moreover, the Bi-PCN-222 nanozyme exhibits the capability to enhance wound healing by increasing fibroblast proliferation and the expression of angiogenesis-related genes such as bFGF, VEGF, and HIF-1α. NSB has introduced novel approaches and methods for the design and advancement of catalytic reactions, holding significant scientific significance and practical value. With the continuous advancement and refinement of nanobiocatalysis technology, we anticipate witnessing further innovation and breakthroughs in the field of biocatalysis.

### 4.4 Imaging

Biomedical imaging technologies play a crucial role in the field of biomedical science, aiding in the observation, diagnosis, monitoring, and evaluation of diseases. For instance, CT imaging permits the examination of internal organ structures [181], ultrasound imaging facilitates the assessment of fetal development [182], and MRI enables the visualization of brain and other soft tissue structures [183]. By employing biomedical imaging technologies, medical professionals can identify diseases at an early stage, thereby improving the effectiveness of treatments.

**Fig. 10:**
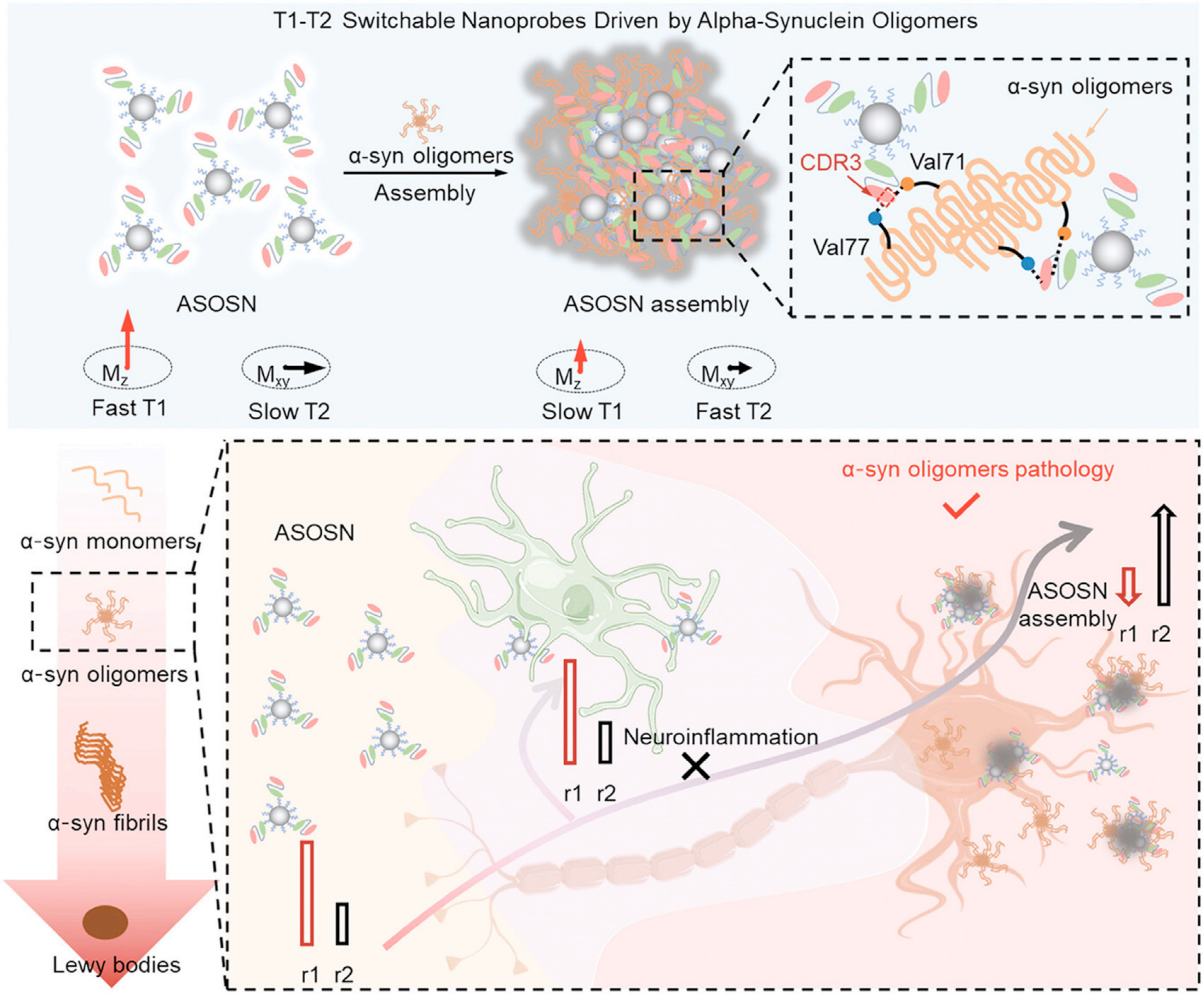
**Schematic depiction of a T1–T2 adaptive ASOSN mechanism driven by α-syn oligomers for the early, accurate diagnosis of PD.** This MRI signal modulator, assisted by ligands targeting α-syn oligomers, enables an adjustable response. As α-syn oligomers bind, the ASOSN's r2 value increases while its r1 value declines, shifting the MRI signal from T1 to T2. In comparison, ASOSN-treated inflammatory areas show minimal signal variation, unlike current T2 MRI agents, allowing ASOSN to distinguish between α-syn oligomers and neuroinflammation. The switchable MRI signaling supports real-time tracking of α-syn oligomers in early PD [185]. Reprinted with permission.

The development of NSB has led to the creation of nano-materials with specific biological recognition functions, enabling high-sensitivity imaging of biomarkers such as disease indicators and cell surface proteins. This advancement has profound implications for disease diagnosis, monitoring, and drug efficacy assessment [184]. For instance, α-synuclein (α-syn) oligomers play a pivotal role in the pathology of Parkinson's disease (PD). The precise identification of α-syn oligomers in vivo presents a hopeful avenue for early and accurate diagnosis of PD. Chen et al. introduced a novel MRI probe (ASOSN) designed for specific MRI, which integrates highly sensitive anti-ferromagnetic nanoparticles functionalized with single-chain variable fragments. This configuration facilitates the recognition and binding of α-syn oligomers (Fig. 10) [185]. The responsive molecular interaction between α-syn oligomers and ASOSN results in a significant increase in relaxivity ratio (r2/r1) and exhibits T2 contrast agent behavior. The accurate and non-invasive imaging of endogenous α-syn oligomers in the mouse brain is made possible by this nanoscale sensor. Importantly, this imaging strategy not only offers a method for early diagnosis of PD but also has the potential to reshape the management landscape of neurodegenerative disorders.

In biological systems, nanoparticles self-assemble in situ to provide targeted imaging and therapeutic effects. Xia et al. reported tyrosine (Tyr)-modified peptide-terminated iodine (I)-doped CuS nanoparticles (CuS-I@P1 NPs) [186]. Once internalized into cells, CuS-I@P1 NPs undergo furin-mediated Tyr-Tyr condensation reactions, ultimately leading to the assembly of CuS-I nanoparticles through dimethyl sulfoxide linkages. The intracellular assembly of CuS-I nanoparticles guided by tumor-specific furin protease demonstrates activatable dual-modal imaging abilities and can be employed for augmented photothermal effects. This facilitates effective tumor therapy and imaging. The strategy of self-catalytic regulation of nanoparticle in situ assembly offers advantages such as heightened specificity and biocompatibility, making it an effective approach for target-specific activation in biomedical therapy and imaging.

Targeting CAR-T cell adoptive transfer has emerged as a highly promising cancer therapy. However, tracking and detecting the distribution of these cells in vivo is also crucial. Stefan et al. utilized Zr-labeled near-infrared fluorescent silica nanoparticles to non-genomically label CAR-T cells, enabling whole-body spatiotemporal cell tracking of human CAR-T cells through a dual-modal PET/near-infrared fluorescence imaging strategy [187]. The application of NSB in imaging offers new tools and methods for biomedical research and clinical diagnostics, potentially advancing the advancement and application of imaging technologies. These holds promise for providing more effective and precise imaging diagnostics and therapeutic interventions for human health.

### 4.5 Biosensor

The application of nanobiocatalysis in biosensors holds significant importance. Nanobiocatalysis allows for the preparation of nanomaterials with specific biomolecular recognition capabilities through surface modification or functionalization. These nanomaterials enable highly sensitive and selective detection of biomolecules, facilitating rapid and accurate detection of trace biomolecules [188,189]. Additionally, nanomaterials serving as carriers for biomolecular binding markers, possessing properties including fluorescence, luminescence, magnetism, and electro-catalytic activity, can achieve real-time monitoring and tracking of biological processes [190].

Currently, a variety of functional and structural nanomaterials, such as nucleic acid nanostructures and protein-nanocomplexes, have been used to construct efficient biosensors [191,192]. These sensors not only detect biomolecules but also support functionalities like localization, quantification, and dynamic monitoring of biomolecules Moreover, nanosensors possess characteristics of portability and small size, allowing integration into platforms such as microfluidic chips and portable devices, thereby enabling rapid, convenient, and cost-effective detection of biomolecules. Presently, sensors associated with nanobiocatalysis can be employed for detecting a wide range of biomolecules, including proteins, nucleic acids, viruses, bacteria, among others [193]. For instance, Chen et al. developed CRISPR-driven photothermal nanotweezers (CRONT) [194]. Under photothermal action, diffusiophoretic force transports nanoparticles to the photothermal center, while the generated heat facilitates the cleavage of DNA-AuNS by the CRISPR/Cas12a complex bound to the target DNA, releasing detection signals and achieving specific DNA detection. This CRISPR-based biosensing strategy exhibits high specificity and sensitivity.

Enhanced by nanoparticles, electrochemical detection offers a direct, swift, and high-throughput strategy for biomolecular recognition and biosensing However, its sensitivity is often limited by the low effective collision frequency. Guo et al. introduced a cascade DNA assembly amplification and CRISPR-responsive DNA hydrogel-based nanoparticle-enhanced electrochemical method to overcome this limitation [195]. Substantial electrochemical responses were observed through the regulation of DNA hydrogel phase transition and the self-electrolysis of silver nanoparticles. They employed target catalytic hairpin assembly and miRNA-induced strand displacement amplification to activate Cas12a/crRNA, which digested the single-stranded regions of the linker in the DNA hydrogel, leading to a gel-to-sol transition. The released AgNPs oxidized upon collision with the electrode, generating instantaneous peaks recorded by the electrode. This entire process amplified the signal for miRNA detection, resulting in ultra-high sensitivity. In practical applications, this system also exhibited high specificity, making it widely applicable in nanobiocatalytic nucleic acid sensors and holding enormous potential for disease diagnosis.

Fareeha et al. designed cerium oxide nanoparticle-based nanozymes with a CRISPR/Cas12a-based gene detection system to develop a dual-mode Salmonella detection platform [192]. Within this framework, the CRISPR/Cas12a system becomes activated upon encountering target DNA, resulting in the cleavage of FAM-labeled probes and subsequent fluorescence emission. Meanwhile, the nanozyme generates a colorimetric response under high-reactivity H_2_O_2_ conditions. The integration of these dual-mode detection methods enhances the specificity and sensitivity of the approach. This biosensor can be applied to the analysis of real food samples (such as chicken, eggs, and beef) for the detection of bacterial concentrations.

In conclusion, the application of NSB in biosensors offers novel methods and pathways for the detection of biomolecules, holding significant scientific significance and practical value. These biosensors, constructed using nanobiotechnology, can find broad applications in clinical diagnostics, environmental monitoring, food safety, and beyond, catering to diverse needs across various domains.

## 5 Conclusion and outlook

NSB integrates synthetic biology and nanotechnology, providing a novel approach to design and construct bioentities and nanomaterials with specific functionalities. The advancement of this field benefits from progress in several aspects, including the understanding of biological engineering in synthetic biology, precise manipulation of nanoscale materials in nanotechnology, and research on interactions between biological and nanomaterials.

In this review, we first introduce the background and motivation of NSB. With deepening understanding of biological systems and nanomaterials, it becomes evident that integrating the two can create numerous new applications and solutions, thereby driving scientific and technological advancements. Secondly, we comprehensively discuss nanocarriers relevant to synthetic biology. Organic, inorganic, and biomimetic nanocarriers each have unique characteristics suitable for various applications. Organic nanocarriers typically possess good biocompatibility and tunability, while inorganic nanocarriers exhibit excellent chemical and physical properties. Biomimetic nanocarriers mimic the structure and function of biological systems, offering stability and biocompatibility for biomimetic material design and construction. The innovation and advancement of these nanocarriers continuously expand the application scope of NSB. The integration of nanomaterials with biological entities such as cells, bacteria, and viruses, has enabled unprecedented functionalities and applications through interactions and synergistic effects. These bio-nano hybrid systems show enormous potential in drug delivery, gene therapy, biocatalysis, bioimaging, biosensing and, promising to drive advancements in related fields.

Future research is likely to focus more on the development of environmentally responsive smart nanosystems. These systems are capable of responding to specific biological signals, such as pH changes, temperature shifts, and ROS levels, allowing for the precise release of drugs or modulation of gene expression at optimal times. Such nanosystems hold great potential in the treatment of cancers, chronic inflammation, and metabolic disorders. With the advancements in artificial intelligence and machine learning, researchers can now automate the design and optimization of genetic circuits, thereby enhancing the efficiency of synthetic biology applications. For instance, machine learning models can be employed to predict the expression levels and interactions of synthetic biological components.

NSB is also expected to play a crucial role in personalized medicine, especially by customizing nanomedicines or synthetic biology solutions to align with the genetic characteristics and disease conditions of individual patients. For example, designing specific nanomedicines tailored to the unique tumor microenvironment of different patients could enhance therapeutic outcomes while minimizing adverse effects.

However, NSB, as an emerging field integrating nanotechnology and synthetic biology, faces several challenges in practical applications. Firstly, the long-term accumulation, metabolism, and excretion of nanomaterials in the body may lead to adverse reactions and toxicity. Optimizing the biocompatibility of nanomaterials, while maintaining therapeutic efficacy and minimizing side effects, is a critical issue that needs to be addressed. Secondly, within the body, nanocarriers often encounter complex and dynamic microenvironments, such as varying pH levels, enzyme activities, and redox states across different tissues. The heterogeneity of these biological environments can make it challenging to predict the behavior of nanomaterials in vivo, potentially affecting their targeted release and functionality. Designing nanomaterials that can better adapt to the intricate in vivo environment, enabling efficient and precise functional regulation, represents a key challenge for NSB.

Additionally, the complexity of nanomaterial synthesis often results in significant batch-to-batch variability and difficulty in standardization, posing obstacles for practical applications. Developing reliable production and quality control methods to enhance product consistency is a crucial area of focus. Furthermore, NSB, which involves genetic, protein, and cellular engineering, may raise ethical concerns, such as issues related to genetic modifications and privacy protection. Given its novelty, existing regulatory frameworks may also lack clear guidelines for evaluating and approving NSB products. These considerations must be thoroughly addressed when advancing the translation of this technology.

In summary, NSB is a vibrant and promising research field. With further understanding of interactions between biological entities and nanomaterials, we can develop more innovative bioapplications, propel technological and scientific advancements, and contribute to addressing major societal challenges.

## Declaration of competing interest

The authors declare that they have no conflicts of interest in this work.
